# MRI‐Derived Lymph Nodes Morphological and Topological Structure (LNs‐MTS) Model for Evaluating Immune Status and Prognosis in Rectal Cancer

**DOI:** 10.1002/advs.202506523

**Published:** 2025-08-31

**Authors:** Yunxiao Liu, Liwen Zhang, Yanfen Cui, Weiyuan Zhang, Hanqing Hu, Shuai Jiao, Jian Ma, Jiale Li, Jun Xiang, Jinna Li, Haiyi Liu, Xiaotang Yang, Jie Tian, Xishan Wang, Guiyu Wang, Xu Guan

**Affiliations:** ^1^ Department of Colorectal Cancer Surgery the Second Affiliated Hospital of Harbin Medical University No. 246 Xuefu Road Harbin Heilongjiang 150086 China; ^2^ CAS Key Laboratory of Molecular Imaging and Beijing Key Laboratory of Molecular Imaging Institute of Automation Chinese Academy of Sciences Beijing 100190 China; ^3^ Department of Radiology Shanxi Cancer Hospital/Shanxi Hospital Affiliated to Cancer Hospital Chinese Academy of Medical Sciences/Cancer Hospital Affiliated to Shanxi Medical University Taiyuan 030013 China; ^4^ Department of Colorectal Surgery Shanxi Province Cancer Hospital/Hospital Afffliated to Cancer Hospital Chinese Academy of Medical Sciences/Cancer Hospital Afffliated to Shanxi Medical University Taiyuan 030013 China; ^5^ Department of Colorectal Surgery National Cancer Center/National Clinical Research Center for Cancer/Cancer Hospital Chinese Academy of Medical Sciences and Peking Union Medical College Beijing 100021 China; ^6^ Department of Radiology the Second Affiliated Hospital of Harbin Medical University No. 246 Xuefu Road Harbin Heilongjiang 150086 China; ^7^ School of Engineering Medicine and School of Biological Science and Medical Engineering Beihang University Beijing 100191 China

**Keywords:** imaging, immune status, lymph nodes, MRI, rectal cancer

## Abstract

Tumor‐draining lymph nodes (LNs) immune status critically influences cancer progression and treatment response, yet reliable non‐invasive assessment remains clinically unavailable. To address this critical gap, an MRI‐derived LNs morphological and topological structure (LNs‐MTS) model is developed to evaluate the immune status of LNs in rectal cancer (RC). Integrating multicenter imaging, transcriptomic, and immunohistochemical data from 1,156 stage I‐II RC patients, enhanced immune activation in non‐metastatic LNs sized ≥0.5 cm and located ≥5 cm from the primary tumor is discovered. Then two quantitative MRI‐derived imaging features across 7,030 radiologically annotated LNs: total LNs volume (tLNV) and total LNs drainage distance (tLND) is developed, forming the basis of the LNs‐MTS model risk subtypes: high‐risk (HRS), moderate‐risk (MRS), and low‐risk (LRS). Patients in the LRS (characterized by large tLNV and distant tLND) show robust immune cell infiltration in the tumor microenvironment and excellent 5‐year survival, whereas HRS (characterized by small tLNV and near tLND) show stromal dominance and poorer prognosis. Clinically, LNs‐MTS offers a more precise and personalized risk assessment than current guideline‐based risk stratification, potentially sparing stage II‐LRS patients from unnecessary adjuvant treatment while identifying stage I‐HRS individuals for more aggressive treatment, providing valuable insights for personalized treatment strategies in RC management.

## Graphical abstract

An MRI‐derived Lymph Nodes morphological and topological structure (LNs‐MTS) model is established to evaluate the immune status of the lymph node system in rectal cancer. It provides a non‐invasive, quantitative approach for prognosis assessment and optimizing adjuvant therapy decisions. This model addresses both over‐treatment and under‐treatment challenges in current practice.

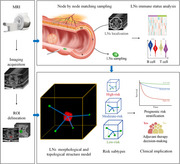

## 1 Introduction

Rectal cancer (RC) remains a leading gastrointestinal malignancy worldwide, with epidemiological data from 2022 reporting over 70 0000 incident cases and 34 0000 deaths.^[^ ^1^ ^]^ Lymph nodes (LNs) evaluation plays a pivotal role in both therapeutic strategy formulation and prognostic prediction for RC. Historically regarded primarily as metastatic reservoirs, LNs are now recognized as immunologically active hubs that coordinate systemic anti‐tumor responses.^[^ ^2^ ^]^ Accumulating evidence demonstrates that anti‐tumor immunity is fundamentally initiated within LNs.^[^ ^3^, ^4^, ^5^, ^6^ ^]^ This paradigm shift underscores the clinical implications of preserving intact immune architecture in non‐metastatic LNs. Consequently, a comprehensive assessment of the LNs system immune status is essential for refining prognostic models and guiding personalized treatment decisions.

Recent advances in LNs biology were highlighted by Paulina et al., who established the paradigm of LNs heterogeneity.^[^ ^7^ ^]^ Their work revealed functional diversity among tumor‐draining LNs, driven by differential anatomical positioning, cellular constituents, and spatial tumor interactions. Supporting this concept, several studies demonstrated that enlarged non‐metastatic LNs exhibit enhanced immune competency compared to their smaller counterparts ^[^ ^8^ ^]^ while distal LNs maintain superior immune activation profiles due to diminished direct tumor suppression.^[^ ^9^ ^]^ These findings collectively underscore the necessity for a multidimensional evaluation of the LNs system, incorporating quantitative metrics (LNs size and count), qualitative immune characterization (individual LN immune status), and spatial architecture (drainage patterns) to achieve a comprehensive immune landscape assessment.

The current evaluation of LNs immune status primarily depends on pathological examination and molecular profiling. While these methods provide valuable insights, they involve complex laboratory procedures and are limited by sampling bias and tissue availability. More critically, such techniques are confined to node‐level analysis, providing only isolated assessments of individual LN or limited LNs subsets. This methodological limitation precludes a comprehensive evaluation of the systemic immune landscape at the patient level. Consequently, the development and validation of innovative non‐invasive methodologies capable of holistically characterizing the LNs system‐wide immune status represents an urgent unmet clinical need.

Magnetic resonance imaging (MRI) has established itself as a high‐resolution modality for LNs evaluation in RC.^[^ ^10^, ^11^ ^]^ Its unique capability to non‐invasively delineate both LNs morphological and spatial topological provides unprecedented opportunities for LNs system evaluation. Notably, while MRI has been extensively utilized for anatomical staging, its potential for functional assessment of LNs immune status remains underexplored. To our knowledge, MRI‐derived non‐invasive methods for assessing the immune status of the LNs system have not been reported, and studies on disclosing the association between LNs imaging features and survival outcomes are lacking.

**Figure 1:**
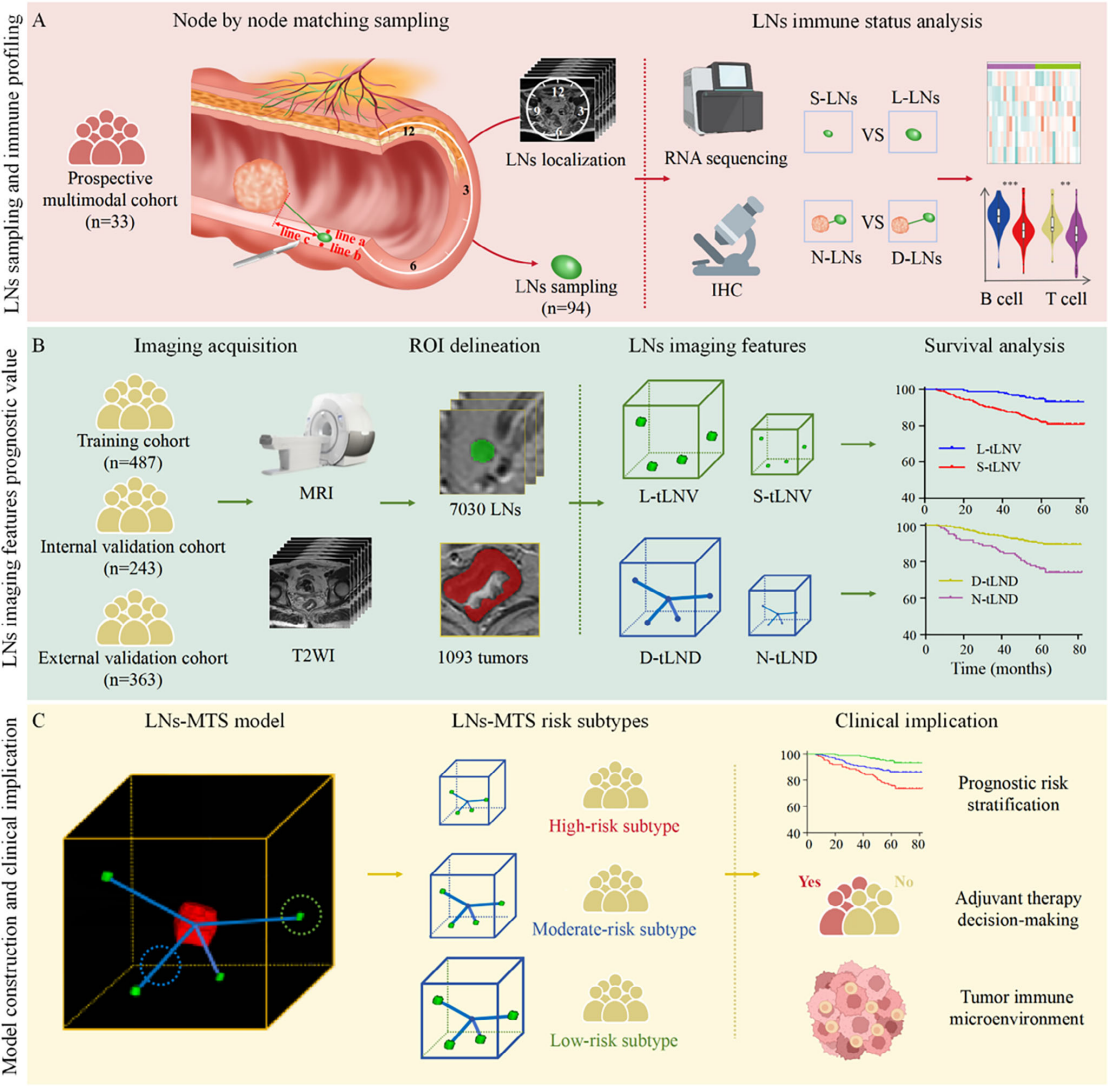
The overall study design. This study establishes a non‐invasive MRI‐based framework for evaluating LNs immune status in rectal cancer. The design encompasses: A) LNs immune status analysis via node‐by‐node matching of MRI‐localized LNs (classified by size [L‐LNs ≥0.5 cm vs S‐LNs <0.5 cm] and distance from tumor [D‐LNs ≥5 cm vs N‐LNs <5 cm]) with paired RNA sequencing and IHC to resolve immune cell distribution; B) MRI‐derived quantitative LNs imaging features, including tLNV and tLND, validated for prognostic utility across cohorts; C) Clinical implication of the LNs‐MTS model, which stratifies patients into HRS, MRS and LRS to guide adjuvant therapy decisions. LNs, lymph nodes; IHC, immunohistochemistry; S‐LNs, small‐LNs; L‐LNs, large‐LNs; N‐LNs, near‐LNs; D‐LNs, distant‐LNs; tLNV, total LNs volume; tLND, total LNs drainage distance; S‐tLNV, small‐tLNV; L‐tLNV, large‐tLNV; N‐tLND, near‐tLND; D‐tLND, distant‐tLND; ROI, region of interest; LNs‐MTS, lymph nodes morphological and topological structure; HRS, high‐risk subtype; MRS, moderate‐risk subtype; LRS, low‐risk subtype.

In this study, we elucidated the immune status of non‐metastatic LNs and delineated significant associations between LNs imaging features and clinical outcomes. We propose an innovative model for evaluating LNs system immune status, namely the LNs morphological and topological structure (LNs‐MTS) model (**Figure** **1**), and identify the relationship between different risk subtypes and their predictive values for prognosis and adjuvant therapy for RC patients.

## 2 Results

### 2.1 Patient Characteristics

The study cohorts comprised 1,156 patients with stage I‐II disease, with a median age of 62 years (IQR: 55–68) and a male predominance (719 males, 62.1%; 437 females, 37.9%). A subset of 63 patients with available genomic and/or IHC data was included for comprehensive immune profiling analysis to evaluate the correlations with imaging features. The patient selection workflow is detailed in Figure S1 (Supporting Information), and **Table** **1** summarizes the clinicopathological characteristics across all patient cohorts.

**Table 1:** Clinicopathological characteristics of patients with rectal cancer across all patient cohorts.

Variables	Training cohort	Internal validation cohort	External validation cohort	Prospective multimodal cohort	RNA sequencing cohort
No. of patients	487	243	363	33	30
Age (n, %)					
≤ 60 years	216 (44.3)	105 (43.2)	162 (44.6)	7 (21.3)	7 (23.3)
> 60 years	271 (55.7)	138 (56.8)	201 (55.4)	26 (78.7)	23 (76.7)
Sex (n, %)					
Male	283 (58.1)	142 (58.4)	249 (68.5)	23 (69.6)	22 (73.3)
Female	204 (41.9)	101 (41.6)	114 (31.5)	10 (30.4)	8 (26.7)
Tumor size (n, %)					
≤ 5 cm	325 (66.7)	152 (62.6)	221 (60.8)	29 (87.9)	19 (63.3)
> 5 cm	162 (33.3)	91 (37.4)	142 (39.2)	4 (12.1)	11 (36.7)
Histology (n, %)					
Adenocarcinoma	479 (98.3)	235 (96.7)	327 (90.1)	32 (97.0)	28 (93.3)
Mucinous and others	8 (1.7)	8 (3.3)	36 (9.9)	1 (3.0)	2 (6.7)
Tumor differentiation (n, %)					
Well/moderate	446 (91.5)	233 (95.8)	337 (92.8)	29 (87.9)	27 (90.0)
Poor /undifferentiated	41 (8.5)	10 (4.2)	26 (7.2)	4 (12.1)	3 (10.0)
LNE (n, %)					
< 12	167 (34.2)	75 (30.8)	73 (20.2)	0 (0.0)	3 (10.0)
≥ 12	320 (65.8)	168 (69.2)	290 (79.8)	33 (100.0)	27 (90.0)
Depth of invasion (n, %)					
T1	15 (3.1)	10 (4.2)	17 (4.7)	1 (3)	0 (0.0)
T2	168 (34.5)	82 (33.7)	82 (22.6)	9 (27.2)	7 (23.3)
T3	182 (37.3)	122 (50.2)	205 (56.5)	22 (66.8)	23 (76.7)
T4	122 (25.1)	29 (11.9)	59 (16.2)	1 (3)	0 (0.0)
TNM staging (n, %)					
I	183 (37.5)	92 (37.9)	99 (27.3)	10 (30.4)	7 (23.3)
II	304 (62.5)	151 (62.1)	264 (72.7)	23 (69.6)	23 (76.7)

LNE, lymph nodes examined.

### 2.2 Immune Status Across LNs Subgroups

**Figure 2:**
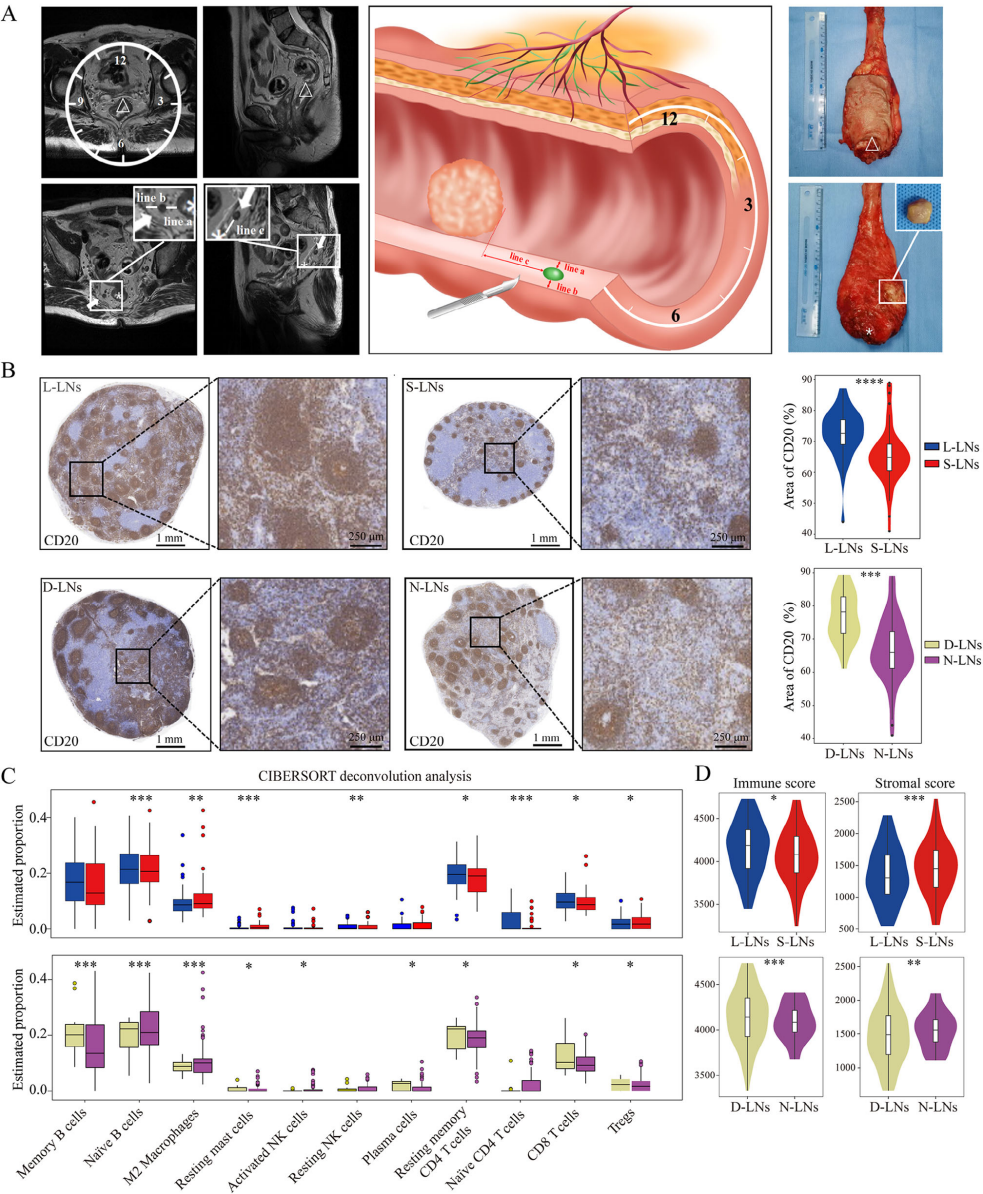
Spatial mapping and functional characterization of LNs in rectal cancer. A) The node‐by‐node matching sampling protocol, where preoperative MRI (left) shows tumor location (△) and LNs (white arrows) with precise spatial measurements including distance to rectal wall (line a), mesorectum (line b), and tumor margin (line c), along with clock position relative to the rectal lumen. The middle panel displays the corresponding 3D reconstruction of LNs spatial relationships, while the right panel demonstrates the pathological correlation with the excised specimen, showing matched LNs (white box) for subsequent molecular analysis. B) Immune cell proportions comparison through IHC analysis, revealing that L‐LNs (n=38) and D‐LNs (n=23) exhibit significantly higher abundance of CD20 B cells compared to their S‐LNs (n=56) and N‐LNs (n=71). C) Characterization of the immune landscape through comparative abundance analysis, demonstrating that L‐LNs and D‐LNs are enriched for tumor‐suppressive immune populations (CD8 T cells and naïve B cells), while S‐LNs and N‐LNs show predominance of tumor‐promoting immune cells (Tregs). D) Quantification of these differences through microenvironment scoring, showing that immunocompetent LNs (L‐LNs and D‐LNs) display significantly elevated immune scores and reduced stromal components, indicating an inverse relationship between immune activation and stromal deposition. Asterisks denote statistical significance levels: ^*^ *P*<0.05, ^**^ *P*<0.01, ^***^ *P*<0.001, ^****^ *P*<0.0001. S‐LNs, small‐LNs; L‐LNs, large‐LNs; N‐LNs, near‐LNs; D‐LNs, distant‐LNs; IHC, immunohistochemistry.

Through integrated multimodal analysis, we systematically characterized the immune status of different LNs. Using preoperative MRI localization, we established precise 3D LNs mapping to enable accurate node‐by‐node correlation between MRI and pathological findings (**Figure** **2**A). Initial IHC evaluation of 94 prospectively collected LNs in the multimodal cohort revealed significant immunological variations, with L‐LNs (n = 38) demonstrating markedly higher abundance of CD4 and CD8 T cells along with CD20 B cells compared to S‐LNs (n = 56) (Figure 2B; Figure S2A, Supporting Information). Similarly, D‐LNs (n = 23) exhibited enhanced T cell (CD3, CD4, and CD8) populations relative to N‐LNs (n = 71) (Figure 2B; Figure S2B, Supporting Information).

To substantiate these findings, we conducted a comprehensive RNA sequencing analysis of LNs specimens, initiating differential expression profiling. Our analysis identified distinct transcriptional patterns that significantly varied according to both LNs size and drainage distance (Figures S3 and S4, Supporting Information). Subsequent immune cell deconvolution revealed quantitative differences in immune cell composition, with D‐LNs and L‐LNs demonstrating substantial enrichment of effector immune populations, particularly CD8 T cells, activated CD4 T cells, and B cells, relative to their counterparts (Figure 2C). Microenvironment scoring algorithms further corroborated these results, showing significantly elevated immune scores and concomitantly reduced stromal components in both D‐LNs and L‐LNs compared to N‐LNs and S‐LNs (Figure 2D; Figure S4E,F, Supporting Information), indicative of a more potent antitumor immune capacity. These differential patterns remained robust across all subgroup analyses (Figures S5 and S6, Supporting Information).

Functional annotation of differentially expressed genes provided mechanistic insights into these immunological disparities. GSEA revealed that L‐LNs were markedly enriched for adaptive immune activation pathways, including Th17 cell differentiation and Th1/Th2 cell polarization (Figure S7A, Supporting Information). Conversely, N‐LNs exhibited preferential activation of tumor‐promoting metabolic pathways, such as AMPK signaling and PPAR‐regulated lipid metabolism (Figure S7B, Supporting Information), suggesting fundamentally divergent functional states between N‐LNs and D‐LNs. Additionally, GO and KEGG enrichment analysis further corroborated these findings, revealing an enrichment of cancer‐related pathways in S‐LNs, indicative of a diminished immune response (Figure S7C,D, Supporting Information). Conversely, immune‐related pathways were significantly enriched in L‐LNs, emphasizing their heightened immune activity and tumor‐suppressive function (Figure S7C,D, Supporting Information). Similarly, N‐LNs displayed an enrichment of cancer‐related pathways, reinforcing their association with lower immune competence (Figure S7E,F, Supporting Information). These results collectively underscore the distinct immunological characteristics of the different LNs subgroups.

### 2.3 Prognostic Value of LNs Imaging Features

**Figure 3:**
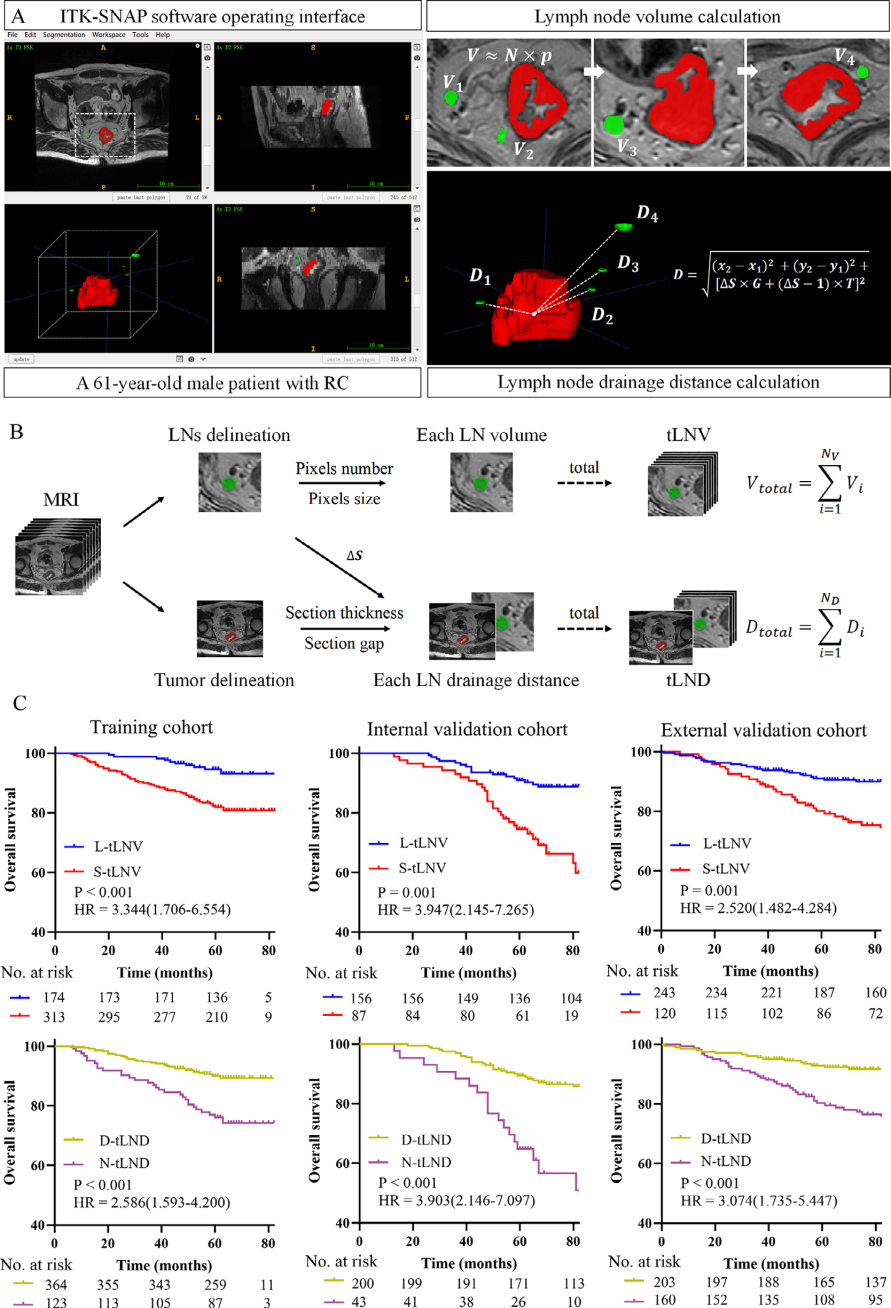
MRI‐derived LNs imaging features predict survival outcomes. A) Feature extraction: tLNV and tLND were quantified from MRI segmentations. B) Computational framework: volume calculations incorporated pixel size/number adjustments; Distance calculations incorporated section thickness/gap adjustments for spatial accuracy. C) Clinical validation: both imaging features independently predicted survival. L‐tLNV and D‐tLND correlated with prolonged overall survival, reflecting robust systemic immunity. RC, rectal cancer; tLNV, total LNs volume; tLND, total LNs drainage distance; S‐tLNV, small‐tLNV; L‐tLNV, large‐tLNV; N‐tLND, near‐tLND; D‐tLND, distant‐tLND; HR, hazard ratio.

Through a comprehensive analysis of 7,030 LNs and 1,093 tumor regions, we developed two quantitative MRI‐derived LNs imaging features (tLNV/tLND). Representative examples of tumor and LNs segmentation, along with feature quantification workflows, were illustrated in **Figure** **3**A,B. We initially assessed the association of LNs imaging features with clinical outcomes (OS and DFS) (Table S1 and Figure S8, Supporting Information). Results showed that the continuous variables tLNV and tLND were predictors of clinical outcomes (Table S2, Supporting Information). In the training cohort, the optimal cutoff values of tLNV and tLND determined by survival analysis were 71.5 mm^3^ and 140.5 mm, respectively (Figure S9, Supporting Information). Therefore, patients were divided into L‐tLNV or S‐tLNV groups and D‐tLND or N‐tLND groups. The associations between the dichotomized tLNV or tLND group and the clinicopathological characteristics within each cohort were summarized in Tables S3–S5 (Supporting Information). We found that patients in the L‐tLNV and D‐tLND groups had better prognosis in the training and validation cohorts (Figure 3C; Figure S10, Supporting Information). Similar results were obtained in subgroup analysis (Figures S11–S14, Supporting Information). Univariate and multivariate analysis also validated that the dichotomized tLNV and tLND remained independent predictive factors for clinical outcomes (Tables S6–S8, Supporting Information; **Table** **2**). These results demonstrated that LNs morphological and topological features provided robust, quantitative biomarkers for predicting survival for RC patients.

**Table 2:** Multivariate Cox regression analysis of LNs‐MTS model imaging features (tLNV and tLND) in patients with rectal cancer.

Variables	Overall survival		Disease‐free survival	
	HR [95%CI]	P	HR [95%CI]	P
Training cohort				
tLNV (S‐ vs L‐tLNV)	2.548 (1.238‐5.245)	0.011	1.954 (1.034‐3.694)	0.039
tLND (N‐ vs D‐tLND)	2.109 (1.251‐3.555)	0.005	1.944 (1.166‐3.243)	0.011
Age (> 60 vs ≤ 60 years)	1.913 (1.121‐3.265)	0.017	1.789 (1.082‐2.959)	0.023
TNM staging (II vs I)	2.718 (1.498‐4.931)	0.001	2.050 (1.208‐3.479)	0.008
Internal validation cohort				
tLNV (S‐ vs L‐tLNV)	3.147 (1.518‐6.522)	0.002	2.982 (1.034‐5.909)	0.002
tLND (N‐ vs D‐tLND)	1.888 (0.927‐3.387)	0.035	2.017 (1.024‐3.974)	0.042
Tumor size (> 5 vs ≤ 5cm)	1.858 (1.020‐3.387)	0.043	1.451 (0.823‐2.556)	0.198
TNM staging (II vs I)	2.527 (1.236‐5.167)	0.011	1.917 (1.025‐3.585)	0.042
External validation cohort				
tLNV (S‐ vs L‐tLNV)	1.662 (0.825‐3.345)	0.018	1.753 (1.122‐3.362)	0.023
tLND (N‐ vs D‐tLND)	2.528 (1.194‐5.355)	0.015	2.387 (1.188‐4.794)	0.014
TNM staging (II vs I)	3.624 (1.627‐8.072)	0.002	3.231 (1.581‐6.592)	0.001

tLNV, total lymph nodes volume; tLND, total lymph nodes drainage distance; S‐tLNV, small‐tLNV; L‐tLNV, large‐tLNV; N‐tLND, near‐tLND; D‐tLND, distant‐tLND; HR, hazard ratio; CI, confidence interval.

### 2.4 Prognostic Performance of the LNs‐MTS Model Risk Subtypes

**Figure 4:**
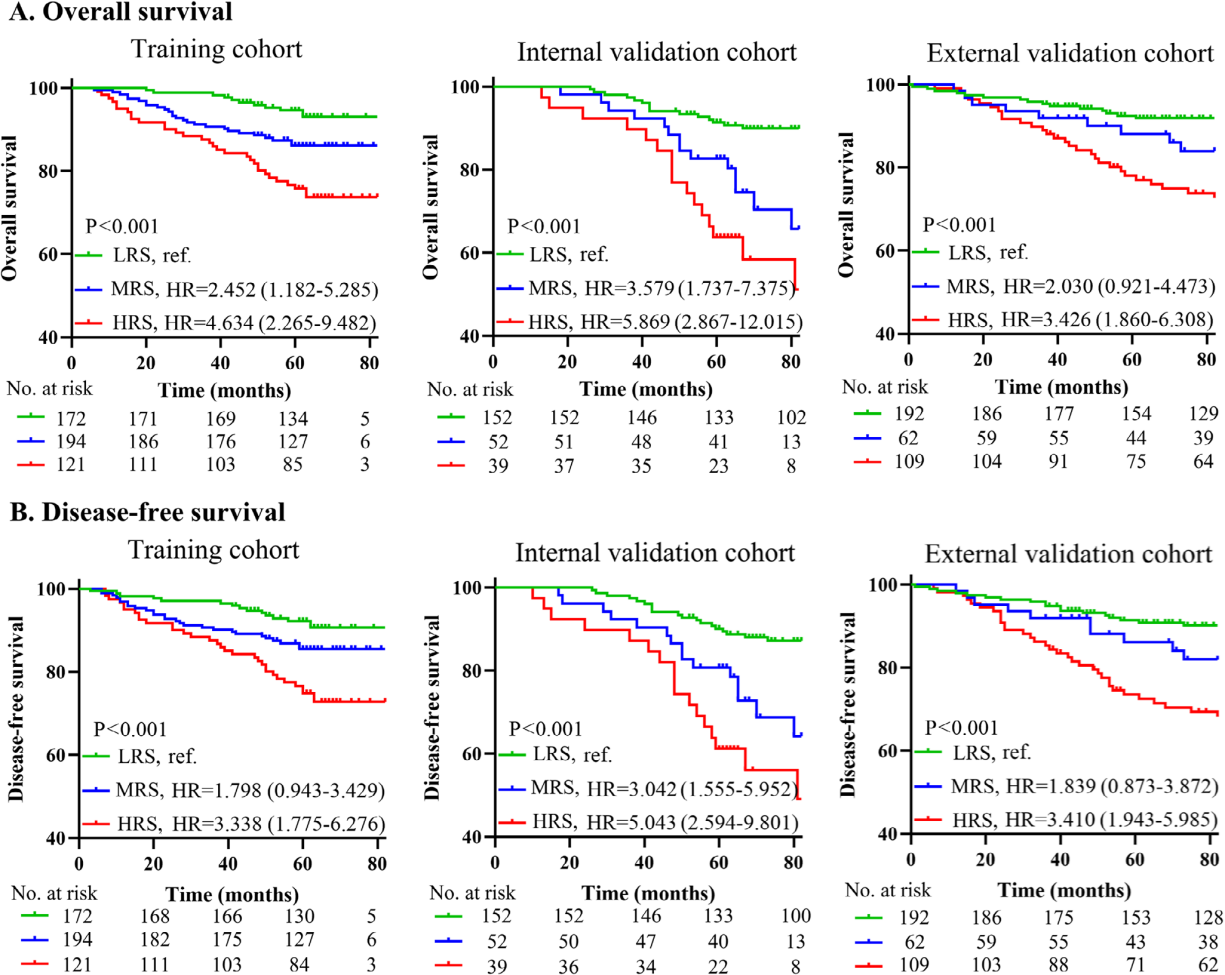
Prognostic stratification by LNs‐MTS model risk subtypes. The LNs‐MTS model integrating tLNV and tLND identified three risk subtypes, including LRS, MRS, and HRS. A) Overall survival comparisons in training and validation cohorts; B) Disease‐free survival comparisons in training and validation cohorts. LRS, low‐risk subtype; MRS, moderate‐risk subtype; HRS, high‐risk subtype; HR, hazard ratio.

The associations between the risk subtypes and baseline characteristics within each cohort were summarized in Tables S9–S11 (Supporting Information). Next, we assessed the prognostic value of the risk subtypes and found that they were strong predictors of OS and DFS in the training cohort (MRS vs LRS, HR = 2.452, 95%CI: 1.182‐5.285; HRS vs LRS, HR = 4.634, 95%CI: 2.265‐9.482, P<0.001), internal validation cohort (MRS vs LRS, HR = 3.579, 95%CI: 1.737‐7.375; HRS vs LRS, HR = 5.869, 95%CI: 2.867‐12.015, P<0.001) and external validation cohort (MRS vs LRS, HR = 2.030, 95%CI: 0.921‐4.473; HRS vs LRS, HR = 3.426, 95%CI: 1.860‐6.308, *P*<0.001) (**Figure** **4**). Among these patients, the highest 5‐year OS and DFS rates were noted in LRS (94.5% and 93.6%, respectively), followed by MRS (88.2% and 87.2%, respectively), and HRS (80.5% and 79.8%, respectively) had the worst 5‐year OS and DFS rates. Multivariate analysis also further validated that the LNs‐MTS model risk subtypes remained independent predictive factors for clinical outcomes (**Table** **3**). Similar results were obtained in subgroup analysis (Figures S15 and S16, Supporting Information).

**Table 3:** Multivariate Cox regression analysis of LNs‐MTS model risk subtypes in patients with rectal cancer.

Variables	Overall survival		Disease‐free survival	
	HR [95%CI]	P	HR [95%CI]	P
Training cohort				
MRS vs LRS	2.398 (1.155‐4.978)	0.019	1.766 (0.925‐3.372)	0.085
HRS vs LRS	5.256 (2.560‐10.793)	<0.001	3.649 (1.932‐6.890)	<0.001
Age (> 60 vs ≤ 60 years)	1.918 (1.124‐3.274)	0.017	1.794 (1.084‐2.996)	0.023
TNM staging (II vs I)	2.722 (1.500‐4.939)	0.001	1.984 (1.168‐3.371)	0.011
Internal validation cohort				
MRS vs LRS	4.268 (2.057‐8.855)	<0.001	3.446 (1.749‐6.749)	<0.001
HRS vs LRS	6.837 (3.327‐14.053)	<0.001	5.550 (2.844‐10.831)	<0.001
Tumor size (> 5 vs ≤ 5cm)	1.826 (1.011‐3.300)	0.046	1.435 (0.821‐2.508)	0.204
TNM staging (II vs I)	2.531 (1.237‐5.178)	0.011	1.925 (1.029‐3.602)	0.040
External validation cohort				
MRS vs LRS	2.104 (0.997‐4.442)	0.051	2.346 (1.062‐5.180)	0.035
HRS vs LRS	4.150 (2.352‐7.323)	<0.001	4.197 (2.268‐7.769)	<0.001
TNM staging (II vs I)	3.625 (1.602‐6.655)	0.001	3.682 (1.655‐8.193)	0.001

HRS, high‐risk subtype; MRS, moderate‐risk subtype; LRS, low‐risk subtype; HR, hazard ratio; CI, confidence interval.

### 2.5 Molecular Characterization of LNs‐MTS Model Risk Subtypes and Tumor Microenvironment

**Figure 5:**
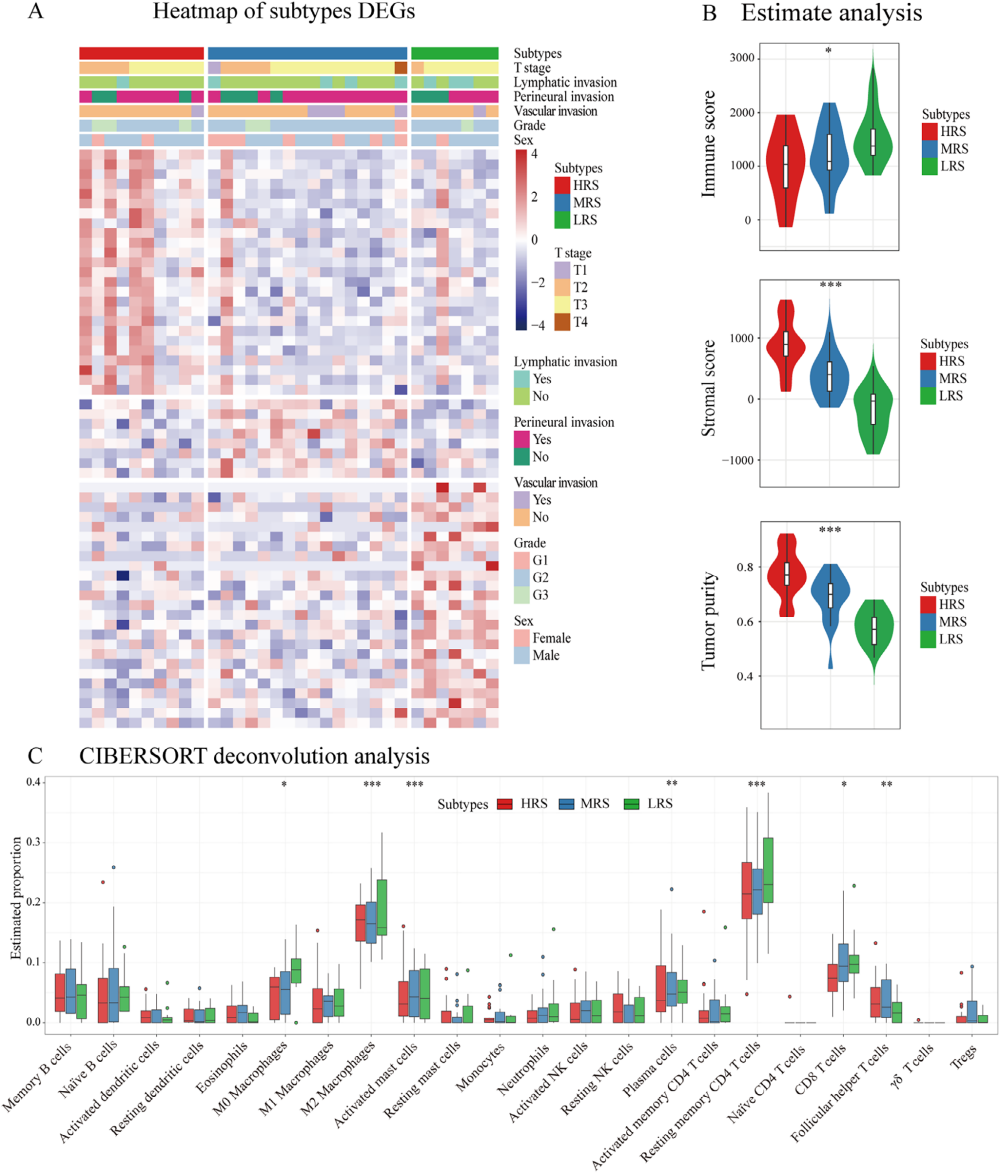
Transcriptomic and immunological profiling among tumor samples of LNs‐MTS model risk subtypes in a prospective multimodal cohort. A) Presents a heatmap of DEGs, revealing distinct transcriptional signatures among subtypes. B) Demonstrates the tumor microenvironment composition through the Estimate algorithm analysis, showing significantly higher immune scores and lower stromal scores in LRS compared to HRS, with intermediate values in MRS. C) CIBERSORT deconvolution analysis is employed to quantify the relative abundances of 22 distinct immune cell populations within the tumor microenvironment, revealing significant differences in immune composition across risk subtypes. Statistical significance: ^*^ *P*<0.05, ^**^ *P*<0.01, ^***^ *P*<0.001. LRS, low‐risk subtype; MRS, moderate‐risk subtype; HRS, high‐risk subtype; DEG, differentially expressed gene.

Through differential expression analysis of tumor tissue gene expression profiles from the prospective multimodal cohort, we identified distinct transcriptional signatures uniquely enriched in each LNs‐MTS risk subtype (**Figure** **5**A). The tumor microenvironment showed progressive immune depletion from LRS (immune‐rich) to HRS (stroma‐dominant) (Figure 5B). CIBERSORT deconvolution analysis demonstrated LRS/MRS enriched cytotoxic CD8 T cells and resting memory CD4 T cells, and HRS was dominated by immunosuppressive M2 macrophages (Figure 5C). These findings explain the prognostic differences, with immune‐active LRS/MRS tumors showing better outcomes than immunosuppressed HRS tumors. The molecular signatures validate LNs‐MTS as a biologically relevant stratification system.

### 2.6 Comparative Prognostic Performance of LNs‐MTS Model

**Figure 6:**
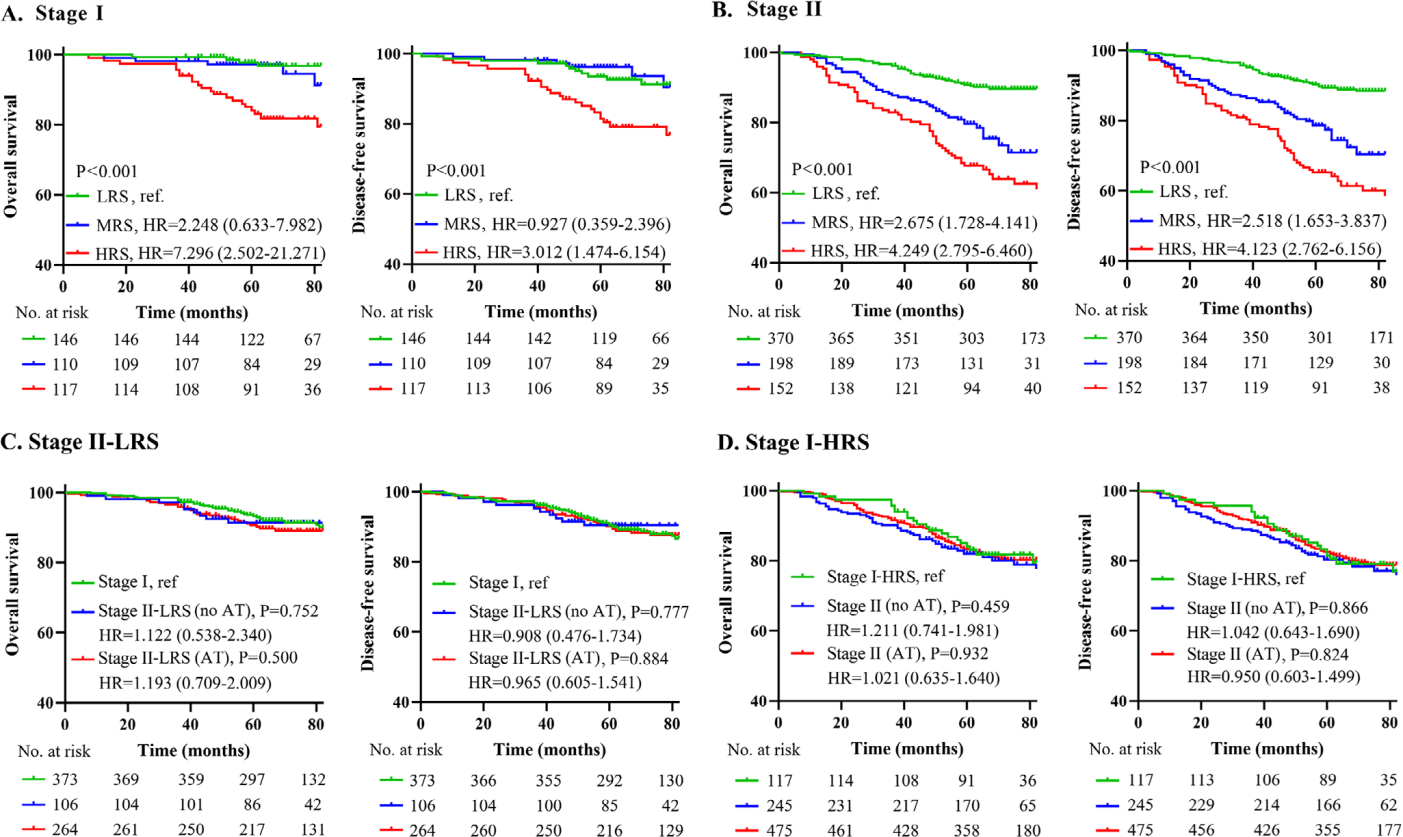
Clinical validation and therapeutic implications of LNs‐MTS risk subtypes. A) Evaluates the model's prognostic performance in stage I patients, revealing distinct survival patterns across risk subtypes. B) Assesses the model's stratification efficacy in stage II patients, demonstrating its ability to identify biologically favorable subtypes. C, D) investigate the differential therapeutic implications, examining the potential for treatment de‐escalation in low‐risk stage II patients and intensification in high‐risk stage I patients. LRS, low‐risk subtype; MRS, moderate‐risk subtype; HRS, high‐risk subtype; AT, adjuvant therapy; HR, hazard ratio.

Comparative analysis demonstrated superior discriminative capacity of the LNs‐MTS risk subtypes over current guideline‐based risk stratification (Figures S17 and S18, Supporting Information). Furthermore, the risk subtypes significantly outperformed the individual LNs imaging features in prognostic prediction. Additionally, discordant risk categorization patterns between the risk subtypes and guideline‐based risk stratification were evident (Figure S19, Supporting Information), suggesting that the LNs‐MTS model risk subtypes may serve as a valuable complementary stratification tool. Subgroup analysis based on TNM staging revealed that for stage I patients, there was no significant difference in clinical outcomes between the LRS and MRS (**Figure** **6**A), likely due to the generally favorable prognosis of stage I patients. However, patients in the HRS showed significantly worse OS and DFS compared to those in the LRS/MRS (Figure 6A). For stage II patients, the LNs‐MTS risk subtypes effectively refined prognosis stratification, providing more granular prognostic insights (Figure 6B).

### 2.7 Therapeutic Decision‐Making Based on LNs‐MTS Risk Subtypes

Our analysis of the LNs‐MTS model identified two clinically actionable subgroups that warrant special consideration in adjuvant treatment planning. Stage II‐LRS patients, constituting 51.4% of stage II cases, demonstrated exceptional 5‐year outcomes (DFS: 90.3%, OS: 90.9%) comparable to those with stage I disease (all P>0.50). Most significantly, adjuvant therapy provided no measurable survival benefit in this subgroup (HR = 1.063, 95%CI: 0.519‐2.175, *P* = 0.87), suggesting that they are ideal candidates for de‐escalation (Figure 6C). Conversely, the stage I‐HRS patients (31.4% of stage I cases) exhibited outcomes mirroring stage II disease (5‐year DFS: 81.5% vs 81.8%) with minimal benefit from standard therapy (HR = 1.004, 95%CI: 0.652‐1.546, P = 0.98), indicating a potential need for treatment intensification (Figure 6D). The ability of the LNs‐MTS model to reclassify ≈44.6% of patients have important clinical implications. By identifying 36.7% of stage II patients who may avoid unnecessary adjuvant therapy and 31.3% of stage I patients who might benefit from more aggressive approaches, this stratification system addresses both over‐treatment and under‐treatment challenges in current practice.

## 3 Discussion

LNs serve as critical secondary lymphoid organs that orchestrate antitumor immunity through adaptive immune surveillance, a fundamental biological process increasingly recognized as a determinant of oncological outcomes.^[^ ^12^, ^13^, ^14^ ^]^ Comprehensive evaluation of LNs system immune status has emerged as an essential component for precise prognostic stratification and therapeutic optimization in cancer management.^[^ ^15^, ^16^, ^17^ ^]^ Our study introduces the LNs‐MTS model, a novel framework that integrates two MRI‐derived quantitative parameters: tLNV, capturing morphological features, and tLND, reflecting topological features. This dual‐parameter approach enables the first comprehensive, non‐invasive assessment of the system‐wide LNs immune status.

Enlargement and numerical expansion of regional LNs represent well‐established morphological manifestations of systemic immune activation within the lymphoid system. Our integrated multimodal analysis, combining RNA sequencing and IHC profiling, demonstrated that L‐LNs exhibited significantly greater activation of immune pathways and higher abundance of effector immune cells compared to S‐LNs, which aligns with previous reports by Ruisch et al.^[^ ^18^ ^]^ and our team's findings.^[^ ^8^ ^]^ Building upon these biological insights, we developed the LNs morphological imaging features (L‐tLNV), which showed a strong association with improved clinical outcomes. This observation extends beyond RC, as evidenced by Chen et al.'s study on triple‐negative breast cancer, which demonstrated that L‐LNs correlate with both enhanced regional antitumor responses and systemic immune activation.^[^ ^19^ ^]^ Similarly, in colorectal cancer, Markl et al. reported improved survival in node‐negative patients with histologically negative L‐LNs,^[^ ^20^ ^]^ supporting the prognostic value of LNs morphological assessment. Our findings establish LNs morphological evaluation as both a biological indicator of immune competence and a clinically relevant prognostic factor. The LNs‐MTS model builds upon this foundation by providing a standardized, quantitative assessment of these critical immunological parameters through non‐invasive imaging.

Our comprehensive analysis of LNs topological features also revealed significant prognostic value that complements morphological assessment. The molecular characterization demonstrated a distinct immunological gradient across the LNs chain, with D‐LNs exhibiting markedly enhanced immune activity and greater activation of critical immune compared to N‐LNs. This spatial immunological pattern reflects the anatomical organization of human LNs as sequential filters, where tumor‐draining sentinel LNs serve as the primary immunological interface with malignant cells.^[^ ^7^ ^]^ The biological basis for this immunological stratification stems from differential exposure to tumor‐derived factors. N‐LNs are exposed to high concentrations of immunosuppressive mediators (VEGF, IL‐3),^[^ ^21^, ^22^, ^23^ ^]^ leading to enhanced regulatory T cell activity, M2 macrophage polarization, and impaired dendritic cell maturation.^[^ ^7^, ^24^ ^]^ D‐LNs maintain functional immune competence due to reduced exposure to direct tumor suppression, preservation of effector T cell populations, and intact antigen presentation capacity.

In the LNs‐MTS framework, the D‐tLND feature reflects either an increased absolute LNs number or a greater proportion of D‐LNs. Both are indicators of systemic immune competence. Conversely, N‐tLND predominance suggests either immune dysfunction or inadequate immune activation. These topological relationships explain the model's strong prognostic performance, with D‐tLND patients showing superior clinical outcomes. By capturing the spatial organization of immune responses, the LN‐MTS model provides unique insights into tumor‐immune interactions that significantly enhance conventional staging systems, offering a robust foundation for personalized therapeutic strategies in RC management.

The LNs‐MTS model fundamentally enhances clinical decision‐making by transcending current conventional TNM staging limitations. Crucially, stage II patients classified as LRS achieved an exceptional 5‐year DFS rate (90.3%) that surpasses standard stage II outcomes (80‐85%) and approaches stage I prognosis, suggesting adjuvant therapy de‐escalation/omission may be clinically warranted for this biologically favorable subgroup. Conversely, the survival rate of patients with stage I‐HRS was significantly reduced, reflecting a high risk of stage II disease. This indicates that despite the early classification of TNM staging, impaired immune capacity may be prone to tumor progression, and adjuvant therapy should still be strongly considered (Figure S20, Supporting Information). This re‐stratification capacity enables precise treatment modulation: de‐escalation/omission for LRS patients and intensification for occult HRS patients. Further clinical trials are needed to prove that.

This study significantly advances the field through three transformative contributions: first, by introducing the first MRI‐derived system‐level assessment model for LNs immune status, which integrates both morphological and topological metrics, we overcome the single‐node limitations of prior pathology‐focused studies. Second, we bridge LNs biology to clinical practice through actionable LNs‐MTS model risk subtypes, demonstrating concrete utility in predicting survival and guiding therapy decisions. Third, our integrated omics validation demonstrates that MRI‐derived risk subtypes directly mirror the tumor immune microenvironment, evidenced by congruent immune activation patterns in tumor RNA sequencing. Critically, this non‐invasive approach eliminates sampling bias while providing real‐time immune assessment unavailable in traditional methods. Though currently limited to non‐metastatic LNs, future integration with deep learning and multimodal data promises expansion to metastatic contexts.

This study has certain limitations. First, variations in MR scanner parameters across different institutions result in imaging diversity. However, we have successfully mitigated the potential adverse effects by undertaking feature calculations that rely on the inherent parameters of the MRI itself. Second, our analysis was confined to mesorectal LNs within the TME field and did not analyze the lateral LNs. Third, the current MR scanning technology has the limitation of being unable to image occult LNs, requiring future integration with functional imaging. Fourth, individual node‐level pathology‐MRI correlation was infeasible in retrospective cohorts due to absent spatial annotations. Future multi‐center prospective cohorts validation remains necessary.

This multicenter study successfully developed and validated the LNs‐MTS model as a novel, non‐invasive method for the comprehensive evaluation of the LNs system immune status in non‐metastatic RC. The model's strong prognostic performance across multiple independent cohorts, coupled with its significant correlations with tumor microenvironment characteristics, establishes its clinical relevance for outcome prediction.

## 4 Experimental Section

### Study Design and Participants

This multicenter study employed a hybrid retrospective‐prospective design, initially screening 1,476 patients with RC from three retrospective cohorts (2011–2020), a prospective multimodal cohort (2023–2024), and an independent RNA sequencing validation cohort (2023–2024). The final analysis included 1,156 eligible patients who met all the inclusion criteria: 1) age 18–75 years; 2) histologically confirmed malignant tumors; 3) pathological stage I‐II disease; 4) R0 resection; 5) preoperative MRI within 7 days before surgery; and 6) ≥ 3 years of follow‐up. The key exclusion criteria were as follows: 1) neoadjuvant therapy; 2) incomplete/inadequate MRI sequences; and 3) missing clinicopathological or follow‐up data. Patients were stratified into a training cohort (n = 487), an internal validation cohort (n = 243), and an independent RNA sequencing validation cohort (n = 30) from Shanxi Cancer Hospital, an external validation cohort (n = 363), and a prospective multimodal cohort (n = 33) from the Second Affiliated Hospital of Harbin Medical University. The study was registered at ClinicalTrials.gov (NCT06319404) and approved by both institutional review boards (YISKY2024‐269; KY2023159), and written informed consent was obtained from all participants.

### Clinical Data Collection and Outcome Measures

Standard clinicopathological variables were systematically extracted from electronic medical records, including demographic characteristics (age, sex), tumor characteristics (size, differentiation grade, histological type), surgical parameters (number of examined LNs, pathological TNM staging), and adjuvant therapy regimens. Primary endpoints were defined: overall survival (OS) as time from curative resection to death from any cause, and disease‐free survival (DFS) as time to tumor recurrence, metastasis, or death. All outcome data were verified through institutional cancer registries, with censoring at last follow‐up for living patients without disease progression.

### Node‐by‐Node Matching Sampling Protocol

A standardized node‐by‐node matching protocol is implemented for LNs sampling and processing to ensure precise spatial correlation between imaging and pathological findings. The methodology involved three critical phases: 1) preoperative MRI‐based spatial mapping utilizing triplanar (axial, sagittal, and coronal) T2‐weighted imaging (T2WI) combined with diffusion‐weighted imaging (DWI) to establish a 3D coordinate system centered on the primary RC lesion, enabling accurate LNs localization and visual reconstruction (Appendix S1, Supporting Information); 2) meticulous intraoperative processing where immediately following resection, mesenteric fat was dissected under sterile conditions with each LN measured before being longitudinally bisected, retaining one half flash‐frozen in liquid nitrogen within 5 min post‐resection for RNA preservation (stored at −80 °C) and the contralateral half fixed in 10% neutral buffered formalin (24–48 h) for routine pathological examination; 3) pathological quality control employing the exclusion criteria: absence of lymphoid tissue on H&E staining, metastatic deposits, or significant processing artifacts, thereby ensuring both the anatomical precision and biological integrity essential for reliable multi‐omic analysis.

### Determination of LNs Classification Thresholds

This study employed rigorously validated thresholds for LNs classification based on both anatomical and immunological criteria. For size stratification, we established a 0.5 cm diameter cutoff to differentiate large LNs (L‐LNs, ≥0.5 cm) from small LNs (S‐LNs, <0.5 cm). This threshold was determined through a systematic analysis of immune marker expression profiles across a clinically relevant size spectrum (0.2–1.0 cm) of 94 LNs in a prospective multimodal cohort, demonstrating maximal discriminatory power for immune activation markers. Similarly, for distance stratification, a 5 cm distance from the primary tumor optimally distinguished distant LNs (D‐LNs, ≥5 cm) from near LNs (N‐LNs, <5 cm), as this boundary showed peak differential expression of immune marker expression profiles across the evaluated ranges (2–10 cm) (Figure S21, Supporting Information). These thresholds showed strong concordance with both existing standards^[^ ^25^, ^26^ ^]^ and our immunohistochemistry (IHC) validation data. Detailed calculation methods are provided in the subhead “Construction of LNs Imaging Features” under the Experimental Section.

### Integrated IHC and Molecular Analysis

For IHC evaluation, LNs specimens underwent standardized processing, including 4% paraformaldehyde fixation (4 °C, 24h), paraffin embedding, and sectioning (4µm thickness). Immunostaining for CD3, CD4, CD8, and CD20 markers was performed using established protocols with DAB chromogenic development (5–10 min), followed by quantitative analysis of immune cell abundance using ImageJ software with uniform threshold settings.

Parallel RNA sequencing analysis was conducted following rigorous quality control: RNA integrity was verified through 1% agarose gel electrophoresis, spectrophotometric quantification (NanoPhotometer), and microfluidic analysis (RNA Nano 6000 Assay Kit, Bioanalyzer 2100).^[^ ^27^ ^]^ Library preparation involved mRNA Capture Beads with Oligo (dT), fragmentation (100–200 nt), cDNA synthesis, and adapter ligation with unique molecular identifiers (UMIs), followed by paired‐end sequencing (Illumina NovaSeq 6000, 150bp reads, 6Gb/sample).^[^ ^28^ ^]^ Bioinformatics processing included adapter trimming and quality filtering (custom Perl scripts), alignment to GRCh38.103 (STAR v2.7.11b),^[^ ^29^ ^]^ and gene quantification (RSEM v1.3.3). Differential expression analysis (|log2FC|>0.05, P<0.05) was performed using limma (v3.52.3), with functional annotation through GO/KEGG enrichment (clusterProfiler v4.4.4)^[^ ^30^, ^31^ ^]^ and GSEA using KEGG pathways. Immune cell deconvolution was conducted via CIBERSORT with LM22 signature matrix (547 genes, 22 cell types) (Appendix S2, Supporting Information),^[^ ^32^ ^]^ complemented by microenvironment scoring (ESTIMATE v1.0.13) to quantify stromal and immune components.^[^ ^33^ ^]^

### MRI Acquisition and Image Analysis Protocol

All patients underwent standardized MRI protocols including T2WI and DWI, with detailed acquisition parameters provided in Appendix S3 and Table S12 (Supporting Information). Image analysis was performed by two radiologists (11 and 12 years of experience in rectal MRI, respectively) using ITK‐SNAP software (v3.8.0) for the volumetric segmentation of primary tumors and perirectal LNs (Appendix S4, Supporting Information). To ensure reproducibility, a consensus‐based delineation protocol was implemented: initial independent annotations were followed by joint review sessions, with any unresolved discrepancies (occurring in ≈15% of cases) adjudicated by a senior radiologist (15 years of experience). This rigorous approach yielded excellent interobserver reliability, as demonstrated by the intraclass correlation coefficients (ICCs) of 0.924 (95% CI: 0.915‐0.933) for tLNV and 0.909 (95% CI: 0.898‐0.918) for tLND measurements (Table S13, Supporting Information). To maintain objectivity while preserving clinical relevance, readers were blinded to all clinicopathological outcomes but were informed of the RC diagnosis to ensure appropriate anatomical evaluations.

### Construction of LNs Imaging Features

In this study, two quantitative MRI‐derived LNs imaging features were developed:

### Construction of LNs Imaging Features—Total LNs Volume (tLNV, mm^3^)

LNV was approximated by calculating the number of pixels in the marked area of the maximum cross‐section. First, the number of pixels in each labeled area was accumulated to obtain the total number of pixels in each LN. Next, the total number of pixels was converted to the actual area based on the size of the pixels. Each LNV was expressed as:

(1) V≈N×p

where *V* represents each LNV, *N* represents the number of pixels, and *p* represents the pixel size. The tLNV is expressed as:

(2) $$V_{\mathrm{total}\;}=\sum_{i=1}^{N_{V}}V_{i}$$

where *V_total_* represents the tLNV, *V_i_* represents the LNV of the *i*‐th LN, and *N_V_* represents the total number of LNs for each patient.

### Construction of LNs Imaging Features—Total LNs Drainage Distance (tLND, mm)

LND was achieved by calculating the Euclidean distance between each LN and the tumor center. First, image processing techniques were used to determine the central location of each LNs and tumor in the MRI section. Second, the MRI section thickness and section gap should be considered. MRI was usually performed in the form of consecutive sections, each of which had a certain thickness and gap between two adjacent sections, which was crucial for the calculation of the 3D distance. Each LND is expressed as follows:

(3) $$D=\sqrt{{\left(x_{2}-x_{1}\right)}^{2}+{\left(y_{2}-y_{1}\right)}^{2}+{\left(z_{2}-z_{1}\right)}^{2}}$$

where (*x*_1_,*y*_1_,*z*_1_) and (*x*_2_,*y*_2_,*z*_2_) represent the 3D coordinates of the LN and tumor, respectively. Suppose that the section thickness of the MRI of a patient is *T* mm and the section thickness is *G* mm, and there is a difference of Δ*S* sections between the LN and the tumor image section. Then, each LND is expressed as:

(4) $$D=\sqrt{{\left(x_{2}-x_{1}\right)}^{2}+{\left(y_{2}-y_{1}\right)}^{2}+{\left[\Delta S\times G+\left(\Delta S-1\right)\times T\right]}^{2}}$$

The tLND is expressed as:

(5) $$D_{\mathrm{total}\;}=\sum_{i=1}^{N_{D}}D_{i}$$

where *D_total_* represents the tLND, *D_i_* represents the LND of the *i*‐th LN, and *N_D_* represents the total number of LNs for each patient.

### Construction of LNs‐MTS Model Risk Subtypes

The LNs‐MTS model was constructed by systematically integrating two quantitative MRI‐derived imaging features: tLNV and tLND. The optimal prognostic thresholds for these imaging features were determined through survival analysis in the training cohort. The optimal thresholds were then applied to categorize the patients into volumetric subgroups (large tLNV [L‐tLNV] vs small tLNV [S‐tLNV]) and spatial subgroups (distant tLND [D‐tLND] vs near tLND [N‐tLND]). These classifications were subsequently combined to create three‐tiered risk subtypes: high‐risk subtype (HRS, identified by concurrent S‐tLNV and N‐tLND), moderate‐risk subtype (MRS, characterized by either L‐tLNV with N‐tLND or S‐tLNV with D‐tLND), and low‐risk subtype (LRS, identified by concurrent L‐tLNV and D‐tLND). The model construction process incorporated rigorous statistical validation to ensure robust discrimination between the risk categories while maintaining clinical interpretability.

### Statistical Analysis

In this study, continuous variables were expressed using appropriate measures of central tendency and dispersion, with normally distributed data reported as mean ± standard deviation (SD) and non‐normally distributed data summarized as median (interquartile range [IQR], Q1–Q3). Between‐group comparisons for continuous variables were conducted using parametric tests or the Mann–Whitney U test. Categorical variables were presented as numbers with percentages and compared using the chi‐square test or Fisher's exact test. Time‐to‐event analysis was performed using Kaplan–Meier methodology with log‐rank testing for survival curve comparisons. Multivariable Cox proportional hazards regression models were employed to assess independent prognostic factors, with results reported as hazard ratios (HR) accompanied by 95% confidence intervals (CI). All statistical analysis conducted in this study were carried out utilizing R software (4.3.1) and GraphPad Prism (10.4.2). All analyses were conducted using two‐tailed tests, with a *P*‐value below 0.05 deemed to indicate statistical significance. Source code is available at https://github.com/dreamenwalker/LN‐MTS.

### Ethics Approval Statement

The ethics committees of the Second Affiliated Hospital of Harbin Medical University (number: YISKY2024‐269) and Shanxi Province Cancer Hospital (number: KY2023159) approved this study.

## Conflict of Interest

The authors declare no conflict of interest.

## Author Contributions

Y.L., L.Z., and Y.C. contributed equally to this study. Y.L., L.Z., and X.G.did conceptualization. Y.L., L.Z., Y.C., and J.X. did the methodology. W.Z., S.J., J.L., J.M., and J.L. curated the data. H.H., W.Z., and H.L. did the investigation. Y.L., J.T., and J.X. did formal analysis. Y.L., L.Z., and Y.C. wrote the original draft. X.Y., J.T., X.W., G.W., and X.G. reviewed and did editing. X.Y., J.T., X.W., G.W., and X.G. supervised the project.

## Supporting information



Supporting Information

## Data Availability

Data are available upon request from the corresponding author.
