# Supplementary material for: MRI‐Derived Lymph Nodes Morphological and Topological Structure (LNs‐MTS) Model for Evaluating Immune Status and Prognosis in Rectal Cancer
Source: Adv Sci (Weinh). 2025 Aug 31;12(39):e06523. doi: 10.1002/advs.202506523 (PMC12533297; doi:10.1002/advs.202506523)
Supplement: Supplementary file 1 — Supporting Information [file ADVS-12-e06523-s001.docx]

**Supplemental material**

**Supplementary methods**

**Appendix S1****. Node-by-node matching sampling protocol**

This protocol details our comprehensive approach for precise correlation between preoperative MRI and pathological specimens in total mesorectal excision (TME) surgeries. The resected TME specimen is conceptualized as a cylindrical structure comprising three concentric anatomical compartments: (1) the inner lumen, (2) surrounding rectal wall, and (3) outer mesorectal fat envelope. To enable accurate spatial mapping, this cylindrical geometry is transformed into a standardized Cartesian coordinate system during pathological processing.

Preoperative MRI serves as the foundation for 3D LN localization through multiplanar reconstruction: ①Sagittal plane: Nodes are categorized by their vertical relationship to the tumor (infratumoral, tumor-level, or supratumoral) based on their position relative to the distal and proximal tumor margins. ②Axial plane: Clockface positioning (12 o'clock ventral to 6 o'clock dorsal) establishes circumferential coordinates relative to the rectal lumen center. Radial depth is measured from the mucosal surface through the mesorectal fat. ③Depth axis: Nodal position within the mesorectum is quantified by orthogonal distance to both the rectal wall and primary tumor.

This triplanar coordinate system generates a patient-specific 3D nodal map. Intraoperatively, the fresh specimen undergoes systematic palpation by both surgeon and pathologist, guided by the radiologist's 3D reconstruction to ensure correspondence between MRI-identified LNs and their pathological counterparts. Each lymph node is then processed with dual preservation: half snap-frozen for molecular analysis and half formalin-fixed for histological evaluation, maintaining perfect spatial annotation throughout the analytical pipeline.

The protocol's rigorous standardization enables unprecedented precision in correlating imaging features with molecular and immunological node characteristics while preserving critical spatial relationships within the mesorectal compartment.

**Appendix S2. Comprehensive immune cell profiling via CIBERSORT deconvolution analysis**

To systematically characterize the immune microenvironment, we performed computational deconvolution of bulk RNA sequencing data using the CIBERSORT algorithm with the LM22 signature matrix. This approach quantifies the relative proportions of 22 functionally distinct immune cell populations within each lymph node sample, encompassing:

1. **B lymphocyte lineage:** Naïve B cells, Memory B cells, Plasma cells;

2. **T cell compartment:** CD8 T cells, CD4 T cell subsets (naïve, resting/activated memory), specialized populations (follicular helper T cells, regulatory T cells [Tregs], γδ T cells);

3. **Innate immune cells:** Natural killer cells (resting/activated), Monocyte-macrophage continuum (M0/M1/M2 polarization states), Dendritic cells (resting/activated), Mast cells (resting/activated), Granulocytes (eosinophils, neutrophils).

The LM22 signature matrix, comprising 547 reference gene expression values, enables sensitive discrimination of these populations even in complex tissue microenvironments. All analysis were conducted with 1000 permutations using default parameters, with results normalized to total immune cell content for cross-sample comparability. This high-resolution immunophenotyping provides critical insights into the functional immune states associated with different LNs subgroups in our study.

**Appendix S3. Standardized multiparametric MRI protocol for rectal cancer evaluation**

All study participants underwent comprehensive rectal MRI examinations performed on 3 Tesla scanners (Philips Achieva or GE Architect systems) equipped with 8-channel phased-array torso coils. The standardized imaging protocol was implemented across all participating centers with the following unified approach:

**Patient preparation and positioning**: Patients were instructed to perform a standardized bowel preparation prior to the MRI procedure. During scanning, patients were positioned supine with a standardized pelvic phased-array coil placement to ensure consistent image quality across all acquisitions.

**Core imaging sequences**: The essential MRI protocol included high-resolution T2-weighted imaging (T2WI) in three orthogonal planes (sagittal, axial, and coronal) along with diffusion-weighted imaging (DWI). The T2WI sequences utilized fast spin-echo acquisition without fat saturation, with oblique axial planes precisely aligned perpendicular to the tumor's longitudinal axis (as determined from sagittal localizers) and oblique coronal planes parallel to the tumor axis. For DWI acquisitions, single-shot echo-planar imaging (SS-EPI) was employed in the transverse plane with motion-probing gradients applied along three orthogonal axes (X, Y, Z), using b-values of 0 and 800-1000 s/mm² and incorporating spectral fat suppression.

**Technical parameters and quality assurance**: All imaging parameters including repetition time (TR), echo time (TE), field of view (FOV), matrix size, and section thickness/gap were systematically recorded and harmonized across different scanner platforms (detailed in Table S1). Following acquisition, images underwent standardized reconstruction and were archived in the picture archiving and communication system (PACS) for subsequent analysis. Rigorous quality control measures were implemented, including real-time assessment of image quality by the performing technologist and secondary verification by study radiologists using predefined quality metrics. Any series demonstrating significant motion artifacts or inadequate coverage were immediately repeated to ensure diagnostic quality.

The multicenter implementation incorporated necessary flexibility to accommodate institutional variations in scanner hardware and software configurations while preserving the essential diagnostic parameters required for consistent tumor staging and LN characterization across all study sites. The resulting images provided the foundation for subsequent comprehensive imaging features calculation in our investigation.

**Appendix S4. Standardized radiological delineation protocol for tumor and LNs characterization**

Our study implemented a rigorous, multi-modal approach, using MRI for image segmentation of rectal tumors and related LNs. All imaging assessments were conducted by radiologists with specialized expertise in rectal cancer and final arbitration by a senior radiologist for discordant interpretations.

For primary tumor evaluation, radiologists performed manual volumetric segmentation on T2WI, delineating the entire tumor extent across sequential axial slices while incorporating both intramural and extramural components. Diffusion-weighted imaging (DWI) with high b-values served as an essential adjunct for tumor and LNs boundary verification, and for detection of suspicious satellite lesions not apparent on anatomical sequences. The segmentation protocol specifically included areas of obvious stromal reaction and tumor-associated desmoplasia while carefully excluding adjacent normal structures.

LNs assessment followed stringent inclusion criteria, with candidate nodes identified in both the mesorectal compartment and along the superior rectal artery vascular pedicle. Only nodes demonstrating a short-axis diameter ≥2 mm on T2WI underwent detailed analysis. For each qualifying node, radiologists contoured the maximal cross-sectional area.

This comprehensive delineation protocol provided the foundation for subsequent feature extraction and predictive modeling, while maintaining direct clinical applicability. The integration of anatomical (T2WI) and functional (DWI) parameters has strongly guaranteed the delineation accuracy of the primary tumor and LNs, with the maximal cross-sectional approach for LNs optimizing the balance between analytical precision and practical implementation in routine practice. All segmentation data underwent systematic archiving in our research PACS with DICOM-RT compatibility to support future analysis and methodologic refinement.

**Supplementary tables**

**Table S1. LNs-MTS model imaging features in training and validation cohorts.**

| **Cohort** | **tLN****V**  **(median, Q1-Q3)** | **tLNV**  **(mean, ±SD)** | **tLND**  **(median, Q1-Q3)** | **tLND**  **(mean, ±SD)** |
| --- | --- | --- | --- | --- |
| **TC** | 51.5 (30.2, 90.0) | 69.2 (64.3) | 218.8 (140.1, 335.2) | 257.3 (175.8) |
| **IVC** | 52.9 (32.0, 80.2) | 62.5 (48.7) | 217.8 (134.1, 335.6) | 245.9 (155.0) |
| **EVC** | 62.3 (34.7, 94.1) | 69.5 (46.1) | 242.2 (157.3, 363.0) | 272.3 (166.6) |

Note: TC, training cohort; IVC, internal validation cohort; EVC, external validation cohort; tLNV, total lymph nodes volume; tLND, total lymph nodes drainage distance;

**Table S2. Univariate cox regression for MRI-derived LNs imaging features and survival outcomes.**

| **Variables** | **Overall survival** | |  | **Disease-free survival** | |
| --- | --- | --- | --- | --- | --- |
|  | **HR (95%CI)** | **P** |  | **HR (95%CI)** | **P** |
| **Training cohort** | | | | | |
| tLNV | 0.986 (0.979-0.993) | <0.001 |  | 0.988 (0.982-0.995) | 0.001 |
| tLND | 0.997 (0.995-0.999) | 0.002 |  | 0.997 (0.996-0.999) | 0.003 |
| **Internal validation cohort** | | | | | |
| tLNV | 0.986 (0.977-0.995) | 0.002 |  | 0.987 (0.979-0.996) | 0.003 |
| tLND | 0.995 (0.992-0.998) | 0.002 |  | 0.996 (0.993-0.999) | 0.003 |
| **External validation cohort** | | | | | |
| tLNV | 0.994 (0.990-0.999) | 0.02 |  | 0.994 (0.990-0.999) | 0.012 |
| tLND | 0.996 (0.994-0.998) | <0.001 |  | 0.996 (0.994-0.998) | <0.001 |

Note: tLNV, total lymph nodes volume; tLND, total lymph nodes drainage distance; HR, hazard ratio; CI, confidence interval.

**Table S****3. Clinicopathological associations of dichotomized tLNV/tLND in training cohort**.

| **Variables** | **Training cohort, n=487** | | | **Training cohort, n=487** | | |
| --- | --- | --- | --- | --- | --- | --- |
|  | **L-tLNV** | **S-tLNV** | **P** | **D-tLND** | **N-tLND** | **P** |
| **Age (n, %)** |  |  | 0.136 |  |  | 0.744 |
| ≤ 60 years | 85 (48.9) | 131 (41.9) |  | 163 (44.8) | 53 (43.1) |  |
| > 60 years | 89 (51.1) | 182 (58.1) |  | 201 (55.2) | 70 (56.9) |  |
| **Sex (n, %)** |  |  | 0.001 |  |  | 0.072 |
| Male | 81 (46.6) | 202 (64.5) |  | 203 (55.8) | 80 (65.0) |  |
| Female | 93 (53.4) | 111 (35.5) |  | 161 (44.2) | 43 (35.0) |  |
| **Tumor size (n, %)** |  |  | 0.001 |  |  | 0.001 |
| ≤ 5 cm | 94 (54.0) | 231 (73.8) |  | 227 (62.4) | 98 (79.7) |  |
| > 5 cm | 80 (46.0) | 82 (26.2) |  | 137 (37.6) | 25 (20.3) |  |
| **Histology (n, %)** |  |  | 0.523 |  |  | 0.104 |
| Adenocarcinoma | 172 (98.9) | 307 (98.1) |  | 360 (98.9) | 119 (96.7) |  |
| Mucinous and Other | 2 (1.1) | 6 (1.9) |  | 4(1.1) | 4 (3.3) |  |
| **Tumor differentiation (n, %)** |  |  | 0.367 |  |  | 0.321 |
| Well /moderate | 162 (93.1) | 284 (90.7) |  | 336(92.3) | 110 (89.4) |  |
| Poor /undifferentiated | 12 (6.9) | 29 (9.3) |  | 28(7.7) | 13 (10.6) |  |
| **LNE (n, %)** |  |  | 0.001 |  |  | 0.001 |
| < 12 | 34 (19.5) | 133 (42.5) |  | 102 (28.0) | 65 (52.8) |  |
| ≥ 12 | 140 (80.5) | 180 (57.5) |  | 262 (72.0) | 58 (47.2) |  |
| **Depth of invasion (n, %)** |  |  | 0.261 |  |  | 0.102 |
| T1 | 3 (1.7) | 12 (3.8) |  | 9 (2.5) | 6 (4.8) |  |
| T2 | 55 (31.6) | 113 (36.2) |  | 117 (32.1) | 51 (41.5) |  |
| T3 | 67 (38.5) | 119 (38.0) |  | 147 (40.4) | 39 (31.7) |  |
| T4 | 49 (28.2) | 69 (22.0) |  | 91 (25.0) | 27 (22.0) |  |
| **TNM staging (n, %)** |  |  | 0.149 |  |  | 0.020 |
| I | 58 (33.3) | 125 (39.9) |  | 126 (34.6) | 57 (46.3) |  |
| II | 116 (66.7) | 188 (60.1) |  | 238 (65.4) | 66 (53.7) |  |

Note: tLNV, total lymph nodes volume; tLND, total lymph nodes drainage distance; S-tLNV, small-tLNV; L-tLNV, large-tLNV; N-tLND, near-tLND; D-tLND, distant-tLND; LNE, lymph nodes examined.

**Table S4. Clinicopathological associations of dichotomized tLNV/tLND in internal validation cohort.**

| **Variables** | **Internal validation cohort**  **n=243** | | | **Internal validation cohort**  **n=243** | | |
| --- | --- | --- | --- | --- | --- | --- |
|  | **L-tLNV** | **S-tLNV** | **P** | **D-tLND** | **N-tLND** | **P** |
| **Age (n, %)** |  |  | 0.912 |  |  | 0.246 |
| ≤ 60 years | 67 (42.9) | 38 (43.7) |  | 83 (41.5) | 22 (51.2) |  |
| > 60 years | 89 (57.1) | 49 (56.3) |  | 117 (58.5) | 21 (48.8) |  |
| **Sex (n, %)** |  |  | 0.094 |  |  | 0.019 |
| Male | 85 (54.5) | 57 (65.5) |  | 110 (55.0) | 32 (74.4) |  |
| Female | 71 (45.5) | 30 (34.5) |  | 90 (45.0) | 11 (25.6) |  |
| **Tumor size (n, %)** |  |  | 0.018 |  |  | 0.314 |
| ≤ 5 cm | 89 (57.1) | 63 (72.4) |  | 128 (64.0) | 24 (55.8) |  |
| > 5 cm | 67 (42.9) | 24 (27.6) |  | 72 (36.0) | 19 (44.2) |  |
| **Histology (n, %)** |  |  | 0.394 |  |  | 0.582 |
| Adenocarcinoma | 152 (97.4) | 83 (95.4) |  | 194 (97.0) | 41 (95.3) |  |
| Mucinous and others | 4 (2.6) | 4 (4.6) |  | 6 (3.0) | 2 (4.7) |  |
| **Tumor differentiation (n, %)** |  |  | 0.696 |  |  | 0..845 |
| Well /moderate | 149 (95.5) | 84 (96.6) |  | 192 (96.0) | 41 (95.3) |  |
| Poor /undifferentiated | 7 (4.5) | 3 (3.4) |  | 8 (4.0) | 2 (4.7) |  |
| **LNE (n, %)** |  |  | 0.038 |  |  | 0.529 |
| < 12 | 41 (26.3) | 34 (39.1) |  | 60 (30.0) | 15 (34.9) |  |
| ≥ 12 | 115 (73.7) | 53 (60.9) |  | 140 (70.0) | 28 (65.1) |  |
| **Depth of invasion (n, %)** |  |  | 0.016 |  |  | 0.01 |
| T1 | 2 (1.3) | 8 (9.2) |  | 5 (2.5) | 5 (11.6) |  |
| T2 | 51 (32.7) | 31 (35.6) |  | 66 (33.0) | 16 (37.2) |  |
| T3 | 85 (54.5) | 37 (42.6) |  | 101 (50.5) | 21 (48.8) |  |
| T4 | 18( 11.5) | 11 (12.6) |  | 28 (14.0) | 1 (4.4) |  |
| **TNM staging (n, %)** |  |  | 0.076 |  |  | 0.176 |
| I | 52 (33.3) | 39 (44.8) |  | 71 (35.5) | 20 (46.5) |  |
| II | 104 (66.7) | 48 (55.2) |  | 129 (64.5) | 23 (53.5) |  |

Note: tLNV, total lymph nodes volume; tLND, total lymph nodes drainage distance; S-tLNV, small-tLNV; L-tLNV, large-tLNV; N-tLND, near-tLND; D-tLND, distant-tLND; LNE, lymph nodes examined.

**Table S5. Clinicopathological associations of dichotomized tLNV/tLND in external validation Cohort.**

| **Variables** | **External validation cohort**  **n=363** | | | **External validation cohort**  **n=363** | | |
| --- | --- | --- | --- | --- | --- | --- |
|  | **L-tLNV** | **S-tLNV** | **P** | **D-tLND** | **N-tLND** | **P** |
| **Age (n, %)** |  |  | 0.141 |  |  | 0.765 |
| ≤ 60 years | 115 (47.3) | 47 (39.2) |  | 92 (45.3) | 70 (43.8) |  |
| > 60 years | 128 (52.7) | 73 (60.8) |  | 111 (54.7) | 90 (56.2) |  |
| **Sex (n, %)** |  |  | 0.172 |  |  | 0.232 |
| Male | 161 (66.3) | 88 (73.3) |  | 134 (66.0) | 115 (71.9) |  |
| Female | 82 (33.7) | 32 (26.7) |  | 69 (34.0) | 45 (28.1) |  |
| **Tumor size (n, %)** |  |  | 0.001 |  |  | 0.002 |
| ≤ 5 cm | 125 (51.4) | 96 (80.0) |  | 109 (53.7) | 112 (70.0) |  |
| > 5 cm | 118 (48.6) | 24 (20.0) |  | 94 (46.3) | 48 (30.0) |  |
| **Histology (n, %)** |  |  | 0.279 |  |  | 0.509 |
| Adenocarcinoma | 216 (88.9) | 111 (92.5) |  | 181 (89.2) | 146 (91.2) |  |
| Mucinous and others | 27 (11.1) | 9 (7.5) |  | 22 (10.8) | 14 (8.8) |  |
| **Tumor differentiation (n, %)** |  |  | 0.861 |  |  | 0.549 |
| Well /moderate | 226 (93.0) | 111 (92.5) |  | 187 (92.1) | 150 (93.8) |  |
| Poor /undifferentiated | 17 (7.0) | 9 (7.5) |  | 16 (7.9) | 10 (6.2) |  |
| **LNE (n, %)** |  |  | 0.006 |  |  | 0.001 |
| < 12 | 39 (16.0) | 34 (28.3) |  | 28 (13.8) | 45 (28.1) |  |
| ≥ 12 | 204 (84.0) | 86 (71.7) |  | 175 (86.2) | 115 (71.9) |  |
| **Depth of invasion (n, %)** |  |  | 0.001 |  |  | 0.061 |
| T1 | 7 (2.9) | 10 (8.3) |  | 7 (3.4) | 10 (6.3) |  |
| T2 | 44 (18.1) | 38 (31.7) |  | 37 (18.2) | 45 (28.1) |  |
| T3 | 143 (58.8) | 62 (51.7) |  | 123 (60.7) | 82 (51.2) |  |
| T4 | 49 (20.2) | 10 (8.3) |  | 36 (17.7) | 23 (14.4) |  |
| **TNM staging (n, %)** |  |  | 0.001 |  |  | 0.007 |
| I | 51 (21.0) | 48 (40.0) |  | 44 (21.7) | 55 (34.4) |  |
| II | 192 (79.0) | 72 (60.0) |  | 159 (78.3) | 105 (65.6) |  |

Note: tLNV, total lymph nodes volume; tLND, total lymph nodes drainage distance; S-tLNV, small-tLNV; L-tLNV, large-tLNV; N-tLND, near-tLND; D-tLND, distant-tLND; LNE, lymph nodes examined.

**Table S6. Univariate cox regression analysis for training cohort.**

| **Variables** | **Overall survival** | |  | **Disease-free survival** | |
| --- | --- | --- | --- | --- | --- |
|  | **HR (95%CI)** | **P** |  | **HR (95%CI)** | **P** |
| tLNV (S- vs. L-tLNV) | 3.344 (1.706-6.554) | <0.001 |  | 2.434 (1.358-4.364) | 0.003 |
| tLND (N- vs. D-tLND) | 2.586 (1.593-4.200) | <0.001 |  | 2.304 (1.445-3.674) | <0.001 |
| Age (> 60 vs. ≤ 60 years) | 2.078 (1.220-3.542) | 0.007 |  | 1.896 (1.148-3.131) | 0.012 |
| Sex (female vs. male) | 1.038 (0.637-1.692) | 0.880 |  | 1.072 (0.672-1.710) | 0.770 |
| Tumor size (> 5 vs. ≤ 5cm) | 1.006 (0.603-1.679) | 0.980 |  | 0.937 (0.571-1.537) | 0.796 |
| Histology | 1.876 (0.458-7.673) | 0.382 |  | 1.693 (0.415-6.912) | 0.463 |
| Tumor differentiation | 1.082 (0.467-2.504) | 0.854 |  | 0.971 (0.421-2.240) | 0.945 |
| LNE (≥ 12 vs. < 12) | 0.815 (0.497-1.335) | 0.415 |  | 0.837 (0.521-1.344) | 0.461 |
| TNM staging (II vs. I) | 2.382 (1.320-4.300) | 0.004 |  | 1.773 (1.050-2.995) | 0.032 |
| Adjuvant therapy (yes vs. no) | 1.472 (0.908-2.388) | 0.117 |  | 1.405 (0.884-2.234) | 0.150 |

Note: tLNV, total lymph nodes volume; tLND, total lymph nodes drainage distance; S-tLNV, small-tLNV; L-tLNV, large-tLNV; N-tLND, near-tLND; D-tLND, distant-tLND; LNE, lymph nodes examined; HR, hazard ratio; CI, confidence interval.

**Table S****7. Univariate cox regression analysis for internal validation cohort.**

| **Variables** | **Overall survival** | |  | **Disease-free survival** | |
| --- | --- | --- | --- | --- | --- |
|  | **HR (95%CI)** | **P** |  | **HR (95%CI)** | **P** |
| tLNV (S- vs. L-tLNV) | 3.947 (2.145-7.265) | <0.001 |  | 3.475 (1.978-6.105) | <0.001 |
| tLND (N- vs. D-tLND) | 3.903 (2.146-7.097) | <0.001 |  | 3.573 (2.021-6.316) | <0.001 |
| Age (> 60 vs. ≤ 60 years) | 1.095 (0.609-1.970) | 0.761 |  | 1.132 (0.650-1.970) | 0.661 |
| Sex (female vs. male) | 0.584 (0.312-1.095) | 0.093 |  | 0.723 (0.408-1.280) | 0.265 |
| Tumor size (> 5 vs. ≤ 5cm) | 1.881 (1.054-3.355) | 0.033 |  | 1.456 (0.843-2.512) | 0.177 |
| Histology | 0.623 (0.086-4.517) | 0.639 |  | 0.545 (0.075-3.943) | 0.548 |
| Tumor differentiation | 0.455 (0.063-3.303) | 0.436 |  | 0.399 (0.055-2.885) | 0.362 |
| LNE (≥ 12 vs. < 12) | 0.833 (0.454-1.528) | 0.555 |  | 0.910 (0.510-1.624) | 0.749 |
| TNM staging (II vs. I) | 2.281 (1.320-4.597) | 0.021 |  | 1.712 (0.928-3.160) | 0.045 |
| Adjuvant therapy (yes vs. no) | 1.868 (0.948-3.678) | 0.071 |  | 1.441 (0.791-2.626) | 0.232 |

Note: tLNV, total lymph nodes volume; tLND, total lymph nodes drainage distance; S-tLNV, small-tLNV; L-tLNV, large-tLNV; N-tLND, near-tLND; D-tLND, distant-tLND; LNE, lymph nodes examined; HR, hazard ratio; CI, confidence interval.

**Table S8. Univariate cox regression analysis for external validation cohort.**

| **Variables** | **Overall survival** | |  | **Disease-free survival** | |
| --- | --- | --- | --- | --- | --- |
|  | **HR (95%CI)** | **P** |  | **HR (95%CI)** | **P** |
| tLNV (S- vs. L-tLNV) | 2.520 (1.482-4.284) | 0.001 |  | 2.566 (1.568-4.199) | <0.001 |
| tLND (N- vs. D-tLND) | 3.074 (1.735-5.447) | <0.001 |  | 3.021 (1.778-5.134) | <0.001 |
| Age (> 60 vs. ≤ 60 years) | 0.814 (0.480-1.382) | 0.446 |  | 0.819 (0.500-1.339) | 0.425 |
| Sex (female vs. male) | 0.683 (0.367-1.272) | 0.229 |  | 0.665 (0.373-1.188) | 0.168 |
| Tumor size (> 5 vs. ≤ 5cm) | 0.626 (0.350-1.119) | 0.114 |  | 0.654 (0.382-1.119) | 0.122 |
| Histology | 1.729 (0.817-3.661) | 0.152 |  | 1.367 (0.650-2.876) | 0.410 |
| Tumor differentiation | 1.643 (0.704-3.837) | 0.251 |  | 1.394 (0.601-3.234) | 0.439 |
| LNE (≥ 12 vs. < 12) | 0.777 (0.423-1.425) | 0.414 |  | 0.895 (0.500-1.601) | 0.708 |
| TNM staging (II vs. I) | 2.768 (1.253-6.118) | 0.012 |  | 2.444 (1.207-4.946) | 0.013 |
| Adjuvant therapy (yes vs. no) | 0.884 (0.494-1.442) | 0.534 |  | 0.962 (0.588-1.576) | 0.878 |

Note: tLNV, total lymph nodes volume; tLND, total lymph nodes drainage distance; S-tLNV, small-tLNV; L-tLNV, large-tLNV; N-tLND, near-tLND; D-tLND, distant-tLND; LNE, lymph nodes examined; HR, hazard ratio; CI, confidence interval.

**Table S9. Baseline characteristics by LNs-MTS risk subtypes in training cohort.**

| **Variables** | **Training cohort, n=487** | | | |
| --- | --- | --- | --- | --- |
|  | **LRS** | **MRS** | **HRS** | **P** |
| **Age (n, %)** |  |  |  | 0.323 |
| ≤ 60 years | 84 (48.8) | 80 (41.2) | 52 (43.0) |  |
| > 60 years | 88 (51.2) | 114 (58.8) | 69 (57.0) |  |
| **Sex (n, %)** |  |  |  | 0.001 |
| Male | 80 (46.5) | 124 (63.9) | 79 (65.3) |  |
| Female | 92 (53.5) | 70 (36.1) | 42 (34.7) |  |
| **Tumor size (n, %)** |  |  |  | <0.001 |
| ≤ 5 cm | 94 (54.7) | 133 (68.6) | 98 (81.0) |  |
| > 5 cm | 78 (45.3) | 61 (31.4) | 23 (19.0) |  |
| **Histology (n, %)** |  |  |  | 0.251 |
| Adenocarcinoma | 170 (98.8) | 192 (99.0) | 117 (96.7) |  |
| Mucinous and Other | 2 (1.2) | 2 (1.0) | 4 (3.3) |  |
| **Tumor differentiation (n, %)** |  |  |  | 0.517 |
| Well /moderate | 160 (93.0) | 178 (91.8) | 108 (89.3) |  |
| Poor /undifferentiated | 12 (7.0) | 16 (8.2) | 13 (10.7) |  |
| **LNE (n, %)** |  |  |  | < 0.001 |
| < 12 | 33 (19.2) | 70 (36.1) | 64 (52.9) |  |
| ≥ 12 | 139 (80.8) | 124 (63.9) | 57 (47.1) |  |
| **Depth of invasion (n, %)** |  |  |  | 0.177 |
| T1 | 3 (1.7) | 6 (3.0) | 6 (5.0) |  |
| T2 | 55 (32.0) | 62 (32.0) | 51 (42.1) |  |
| T3 | 66 (38.4) | 82 (42.3) | 38 (31.4) |  |
| T4 | 48 (27.9) | 44 (22.7) | 26 (21.5) |  |
| **TNM staging (n, %)** |  |  |  | 0.043 |
| I | 58 (33.7) | 68 (35.1) | 57 (47.1) |  |
| II | 114 (66.3) | 126 (64.9) | 64 (52.9) |  |

Note: HRS, high-risk subtype; MRS, moderate-risk subtype; LRS, low-risk subtype; LNE, lymph nodes examined.

**Table S10. Baseline characteristics by LNs-MTS risk subtypes in internal validation cohort.**

| **Variables** | **Internal validation cohort, n=243** | | | |
| --- | --- | --- | --- | --- |
|  | **LRS** | **MRS** | **HRS** | **P** |
| **Age (n, %)** |  |  |  | 0.466 |
| ≤ 60 years | 65 (42.8) | 20 (38.5) | 20 (51.3) |  |
| > 60 years | 87 (57.2) | 32 (61.5) | 19 (48.7) |  |
| **Sex (n, %)** |  |  |  | 0.080 |
| Male | 81 (53.3) | 33 (63.5) | 28 (71.8) |  |
| Female | 71 (46.7) | 19 (36.5) | 11 (28.2) |  |
| **Tumor size (n, %)** |  |  |  | 0.106 |
| ≤ 5 cm | 89 (58.6) | 39 (75.0) | 24 (61.5) |  |
| > 5 cm | 63 (41.4) | 13 (25.0) | 15 (38.5) |  |
| **Histology (n, %)** |  |  |  | 0.132 |
| Adenocarcinoma | 149 (98.0) | 48 (92.3) | 38 (97.4) |  |
| Mucinous and Other | 3 (2.0) | 4 (7.7) | 1 (2.6) |  |
| **Tumor differentiation (n, %)** |  |  |  | 0.737 |
| Well /moderate | 146 (96.1) | 49 (94.2) | 38 (97.4) |  |
| Poor /undifferentiated | 6 (3.9) | 3 (5.8) | 1 (2.6) |  |
| **LNE (n, %)** |  |  |  | 0.232 |
| < 12 | 41 (27.0) | 19 (36.5) | 15 (38.5) |  |
| ≥ 12 | 111 (73.0) | 33 (63.5) | 24 (61.5) |  |
| **Depth of invasion (n, %)** |  |  |  | 0.006 |
| T1 | 2 (1.3) | 3 (5.8) | 5 (12.8) |  |
| T2 | 49 (32.2) | 19 (36.5) | 14 (35.9) |  |
| T3 | 83 (54.7) | 20 (38.5) | 19 (48.7) |  |
| T4 | 18 (11.8) | 10 (19.2) | 1 (2.6) |  |
| **TNM staging (n, %)** |  |  |  | 0.193 |
| I | 51 (33.6) | 21 (40.4) | 19 (48.7) |  |
| II | 101 (66.4) | 31 (59.6) | 20 (51.3) |  |

Note: HRS, high-risk subtype; MRS, moderate-risk subtype; LRS, low-risk subtype; LNE, lymph nodes examined.

**Table S11. Baseline characteristics by LNs-MTS risk subtypes in external validation cohort.**

| **Variables** | **External validation cohort, n=363** | | | |
| --- | --- | --- | --- | --- |
|  | **LRS** | **MRS** | **HRS** | **P** |
| **Age (n, %)** |  |  |  | 0.563 |
| ≤ 60 years | 89 (46.4) | 29 (46.8) | 44 (40.4) |  |
| > 60 years | 103 (53.6) | 33 (53.2) | 65 (59.6) |  |
| **Sex (n, %)** |  |  |  | 0.069 |
| Male | 129 (67.2) | 37 (59.7) | 83 (76.1) |  |
| Female | 63 (32.8) | 25 (40.3) | 26 (23.9) |  |
| **Tumor size (n, %)** |  |  |  |  |
| ≤ 5 cm | 100 (52.1) | 34 (54.8) | 87 (79.8) | <0.001 |
| > 5 cm | 92 (47.9) | 28 (45.2) | 22 (20.2) |  |
| **Histology (n, %)** |  |  |  | 0.222 |
| Adenocarcinoma | 172 (89.6) | 53 (85.5) | 102 (93.6) |  |
| Mucinous and Other | 20 (10.4) | 9 (14.5) | 7 (6.4) |  |
| **Tumor differentiation (n, %)** |  |  |  | 0.353 |
| Well /moderate | 179 (93.2) | 55 (88.7) | 103 (94.5) |  |
| Poor /undifferentiated | 13 (6.8) | 7 (11.3) | 6 (5.5) |  |
| **LNE (n, %)** |  |  |  | 0.001 |
| < 12 | 24 (12.5) | 19 (30.6) | 30 (27.5) |  |
| ≥ 12 | 168 (87.5) | 43 (69.4) | 79 (72.5) |  |
| **Depth of invasion (n, %)** |  |  |  |  |
| T1 | 5 (2.6) | 4 (6.5) | 8 (7.3) | 0.007 |
| T2 | 32 (16.7) | 17 (27.4) | 33 (30.3) |  |
| T3 | 119 (62.0) | 28 (45.1) | 58 (53.2) |  |
| T4 | 36 (18.7) | 13 (21.0) | 10 (9.2) |  |
| **TNM staging (n, %)** |  |  |  | 0.001 |
| I | 37 (19.3) | 21 (33.9) | 41 (37.6) |  |
| II | 155 (80.7) | 41 (66.1) | 68 (62.4) |  |

Note: HRS, high-risk subtype; MRS, moderate-risk subtype; LRS, low-risk subtype; LNE, lymph nodes examined.

**Table S12. MRI acquisition parameters across study cohorts.**

| **Cohort** | **Scanner** | **Sequence** | **TR**  **(msec)** | **TE**  **(msec)** | **Section Thickness (mm)** | **Section Gap (mm)** | **Matrix** | **Pixel**  **(mm^2^)** | **FOV**  **(cm)** | **NSA** |
| --- | --- | --- | --- | --- | --- | --- | --- | --- | --- | --- |
| **TC** | Philips3.0-T (Achieva) | T2WI | 3000 | 80 | 3 | 0.3 | 300×233 | 0.35×0.35 | 18×18 | 3 |
| **IVC** | Philips3.0-T (Achieva) | T2WI | 3000 | 80 | 3 | 0.3 | 300×233 | 0.35×0.35 | 18×18 | 3 |
| **EVC** | GE3.0-T (Architect) | T2WI | 5476 | 120 | 3.6 | 0.3 | 384×256 | 0.2×0.2 | 20×20 | 3 |
| **PC** | GE3.0-T  (Architect) | T2WI | 5373 | 96 | 4 | 1 | 320×320 | 0.54×0.54 | 24×24 | 3 |
| **SC** | Philips3.0-T (Achieva) | T2WI | 3000 | 80 | 3 | 0.3 | 300×233 | 0.35×0.35 | 18×18 | 3 |

Note:TC, training cohort; IVC, internal validation cohort; EVC, external validation cohort; PC, prospective cohort; SC, sequencing cohort; T2WI, T2-weighted imaging; TE, echo time; TR, repetition time; FOV, field of view, NSA, number of signal average.

**Table S13. Interobserver variability of MRI-derived LNs imaging features.**

| **Variables** | **ICC (C, 1)** | **95%CI** |
| --- | --- | --- |
| tLNV | 0.924 | 0.915-0.933 |
| tLND | 0.909 | 0.898-0.918 |

Note: tLNV, total lymph nodes volume; tLND, total lymph nodes drainage distance; ICC (C, 1): intraclass correlation coefficients (two-way randomymixed, single consisiency).

**Supplementary figures**

**Figure S1. The study flow chart.** A hybrid retrospective-prospective analysis of 1,156 non-metastatic rectal cancer patients from five independent cohorts was performed. Prospective multimodal cohort (n=33) provided matched RNA-seq/IHC data for LNs immune profiling, with an independent sequencing validation cohort (n=30) confirming these molecular findings. The study workflow of retrospective cohorts highlights quality control, ensuring robust validation of the LNs-MTS model in training (n=487), internal (n=243), and external (n=363) validation cohorts.

**
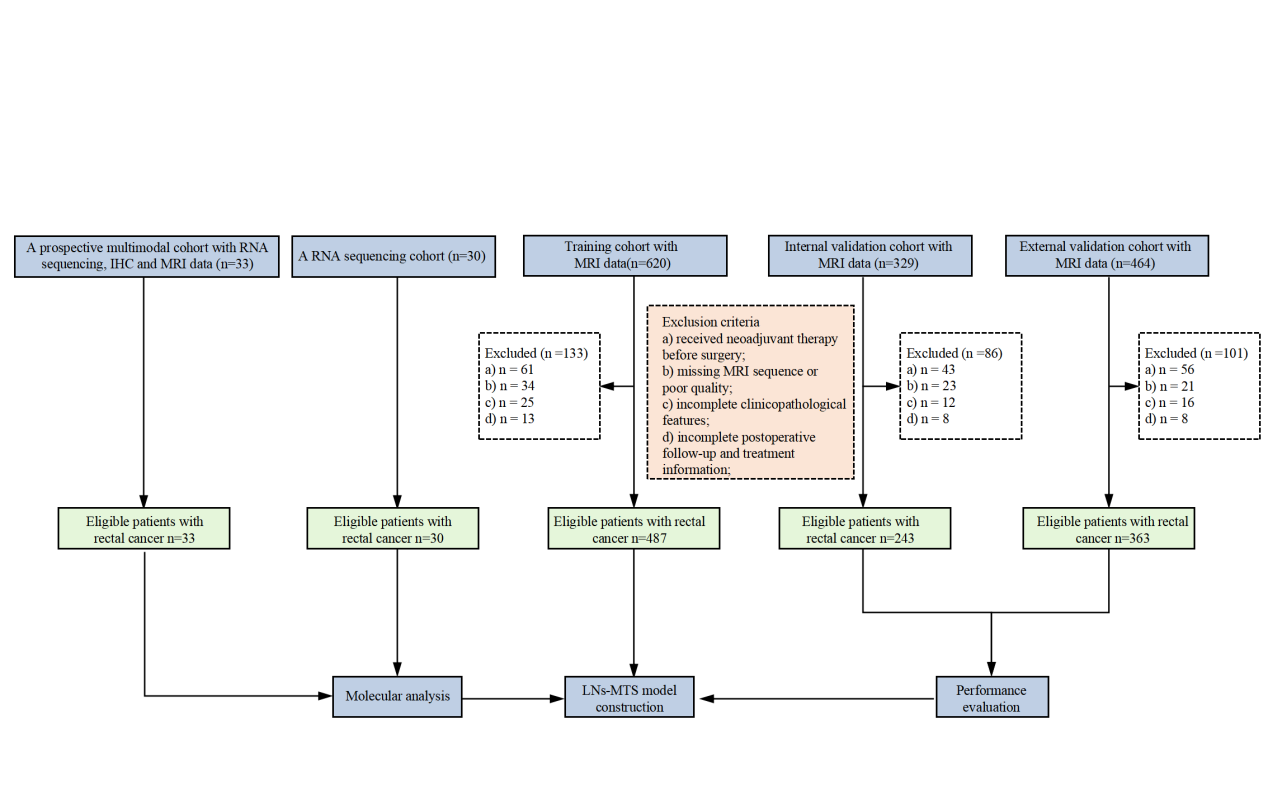
**

**Figure S2. Comparative analysis of immune cell distribution in LNs subgroups based on IHC**. (A) Compares immune cell proportions between L-LNs and S-LNs. (B) Examines differences between D-LNs and N-LNs. The boxplots visualize the distribution of immune cell markers, where the central line represents the median value, box boundaries indicate the interquartile range, and whiskers show the data variability. Asterisks denote statistically significant differences between comparison groups (p<0.05). Statistical significance thresholds: *P<0.05; **P<0.01; ***P<0.001; ****P<0.0001. S-LNs, small-LNs; L-LNs, large-LNs; N-LNs, near-LNs; D-LNs, distant-LNs.

**
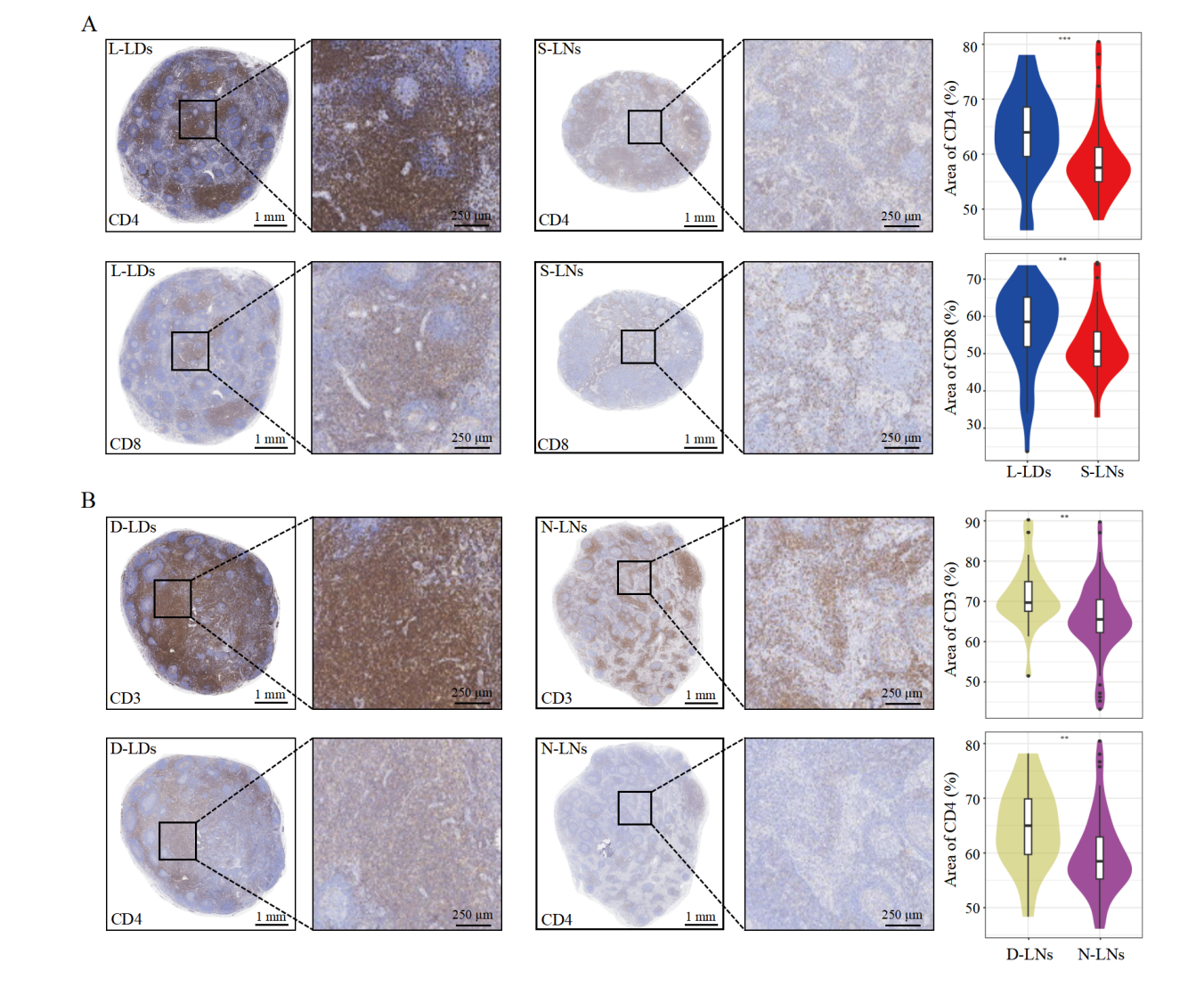
**

**Figure S3. Transcriptomic profiling of LNs subgroups in prospective multimodal cohort. (A)** A volcano plot illustrating the DEGs between D-LNs and N-LNs, highlighting genes with significant upregulation and downregulation. **(B)** A volcano plot depicting the differentially expressed genes between L-LNs and S-LNs, providing insights into key transcriptional differences between these subgroups. **(C)** A heatmap visualizing the expression patterns of selected genes between D-LNs and N-LNs, capturing distinct gene expression signatures across these LNs subgroups. **(D)** A heatmap showcasing the expression differences of selected genes between L-LNs and S-LNs, offering a comparative view of gene regulation within these LNs subgroups. S-LNs, small-LNs; L-LNs, large-LNs; N-LNs, near-LNs; D-LNs, distant-LNs.


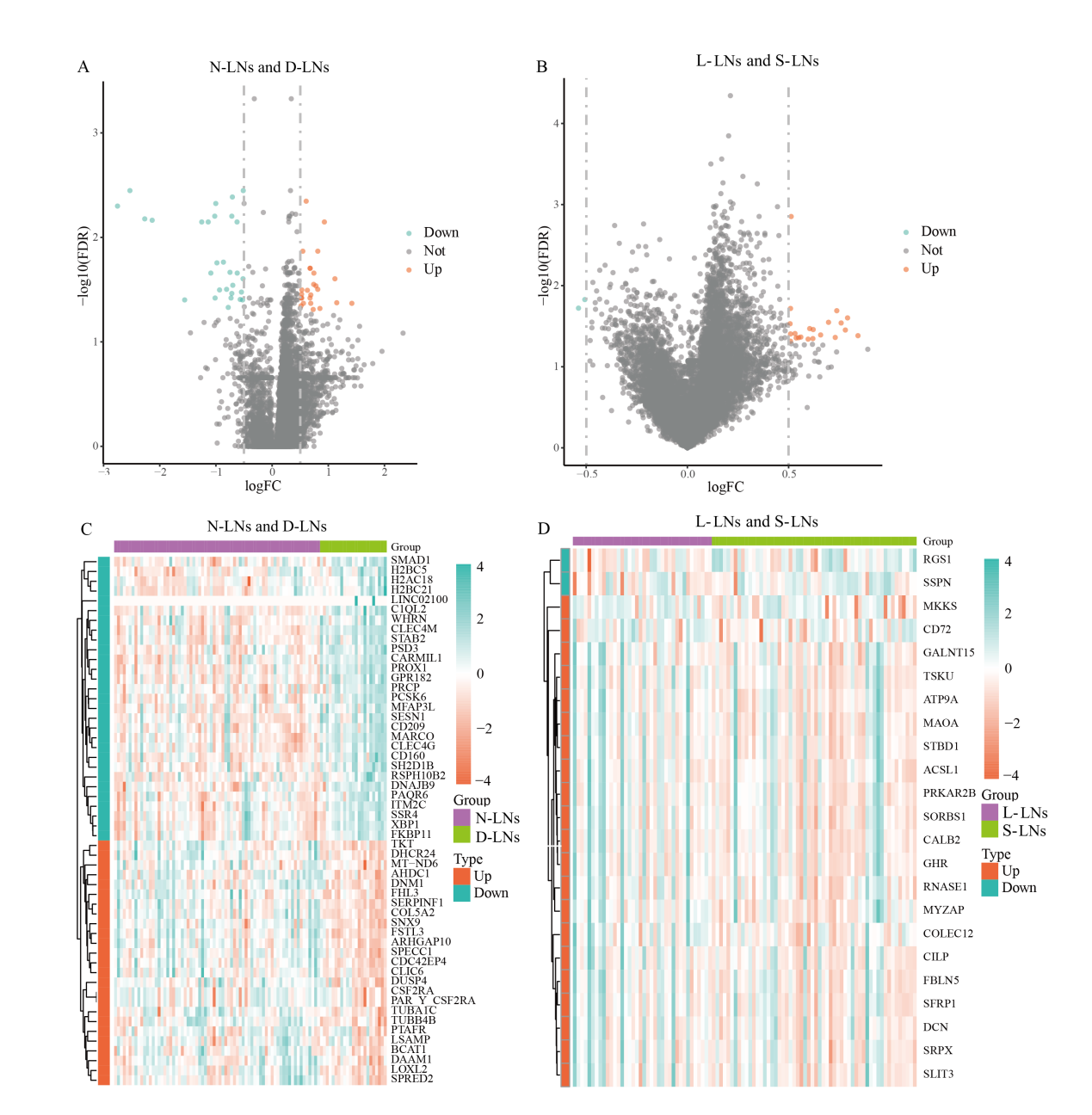


**Figure S4. Transcriptomic profiling of LNs subgroups in sequencing cohort.** (A) Volcano plot of DEGs between N-LNs and D-LNs, highlighting genes with significant upregulation and downregulation. (B) Volcano plot of DEGs between L-LNs and S-LNs. (C) Heatmap of DEGs between D-LNs and N-LNs. (D) Heatmap of DEGs between L-LNs and S-LNs. (E) Comparison of immune and stromal scores between D-LNs and N-LNs (F) Comparison of immune and stromal scores between L-LNs and S-LNs. S-LNs, small-LNs; L-LNs, large-LNs; N-LNs, near-LNs; D-LNs, distant-LNs.

**
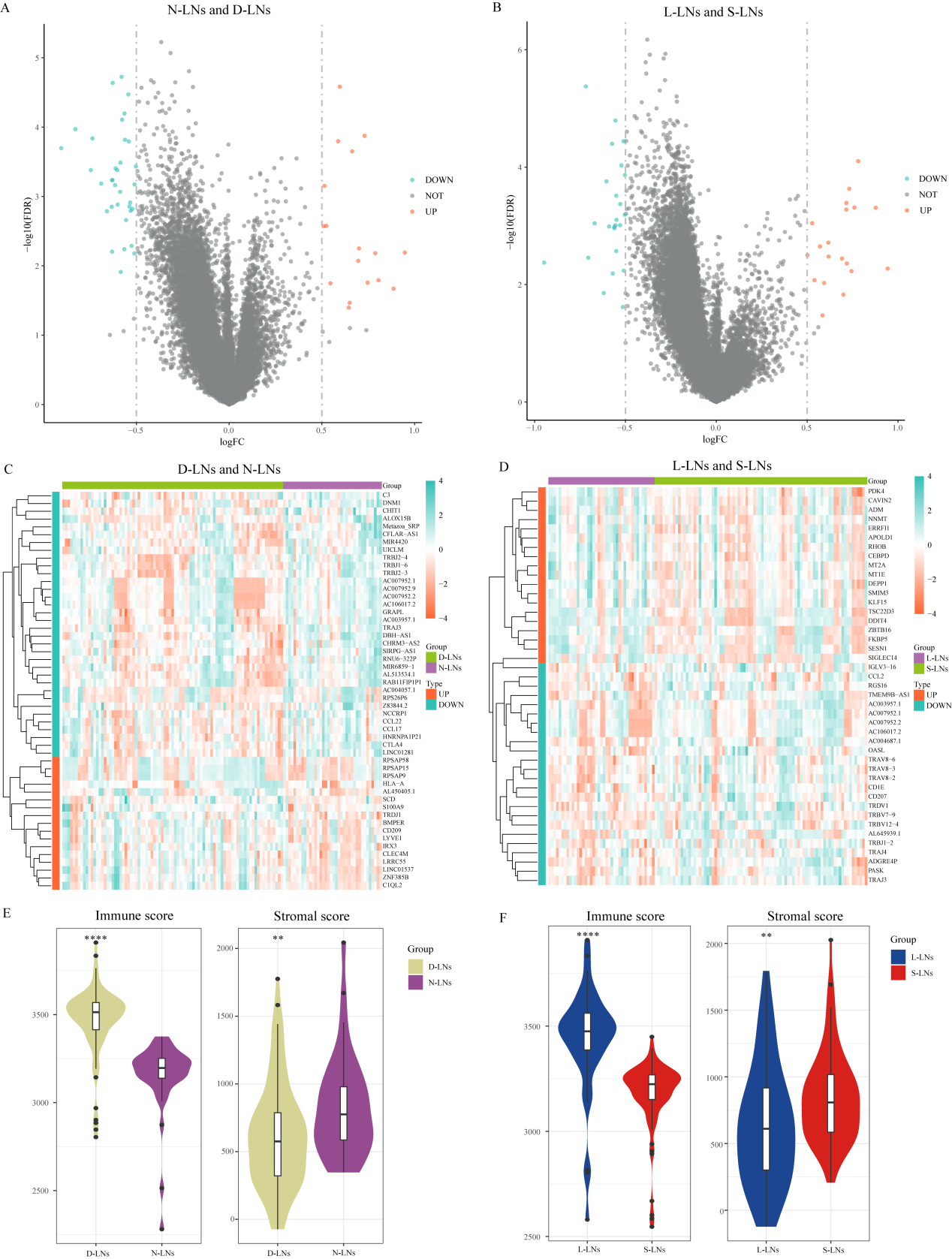
**

**Figure S5. Integrated transcriptomic analysis of LNs subgroups in prospective multimodal cohort. (A)** A volcano plot and heatmap illustrating gene expression differences between L-LNs and S-LNs within the N-LNs subgroup, highlighting key differentially expressed genes. **(B)** A volcano plot and heatmap depicting the gene expression variations between L-LNs and S-LNs within the D-LNs subgroup, providing insights into transcriptional differences in this context. **(C)** A volcano plot and heatmap showcasing the differential gene expression patterns between D-LNs and N-LNs within the L-LNs subgroup, revealing distinct molecular signatures. **(D)** A volcano plot and heatmap representing the gene expression differences between D-LNs and N-LNs within the S-LNs subgroup, emphasizing key transcriptional changes in this comparison. S-LNs, small-LNs; L-LNs, large-LNs; N-LNs, near-LNs; D-LNs, distant-LNs.

**
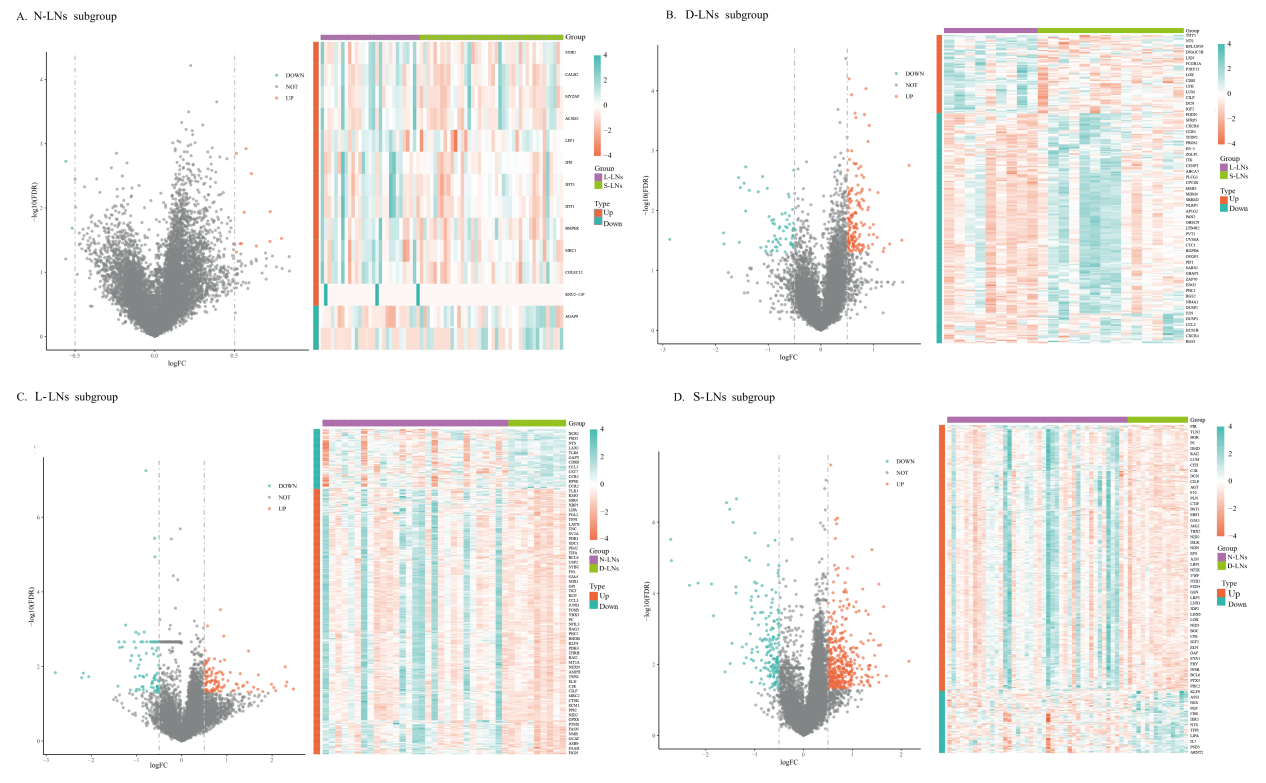
**

**Figure S6. Comprehensive immune landscape characterization across LNs subgroups in prospective multimodal cohort. (A)** Comparative analysis of microenvironment scoring and immune cell abundance between L-LNs and S-LNs within the N-LNs subgroup, highlighting distinct immunological landscapes. **(B)** Microenvironment scoring an immune cell abundance between L-LNs and S-LNs within the D-LNs subgroup, providing insights into immune microenvironment variations. **(C)** Microenvironment scoring analysis comparing D-LNs and N-LNs within the L-LNs subgroup, revealing differences in immune cell composition and potential immunological implications. **(D)** Evaluation of microenvironment scoring and immune cell abundance differences between D-LNs and N-LNs within the S-LNs subgroup, illustrating key immunological divergences. Each panel integrates multiple immune cell type analysis, with asterisks denoting statistical significance (*P<0.05, **P<0.01, ***P<0.001, ****P<0.0001). S-LNs, small-LNs; L-LNs, large-LNs; N-LNs, near-LNs; D-LNs, distant-LNs.

**
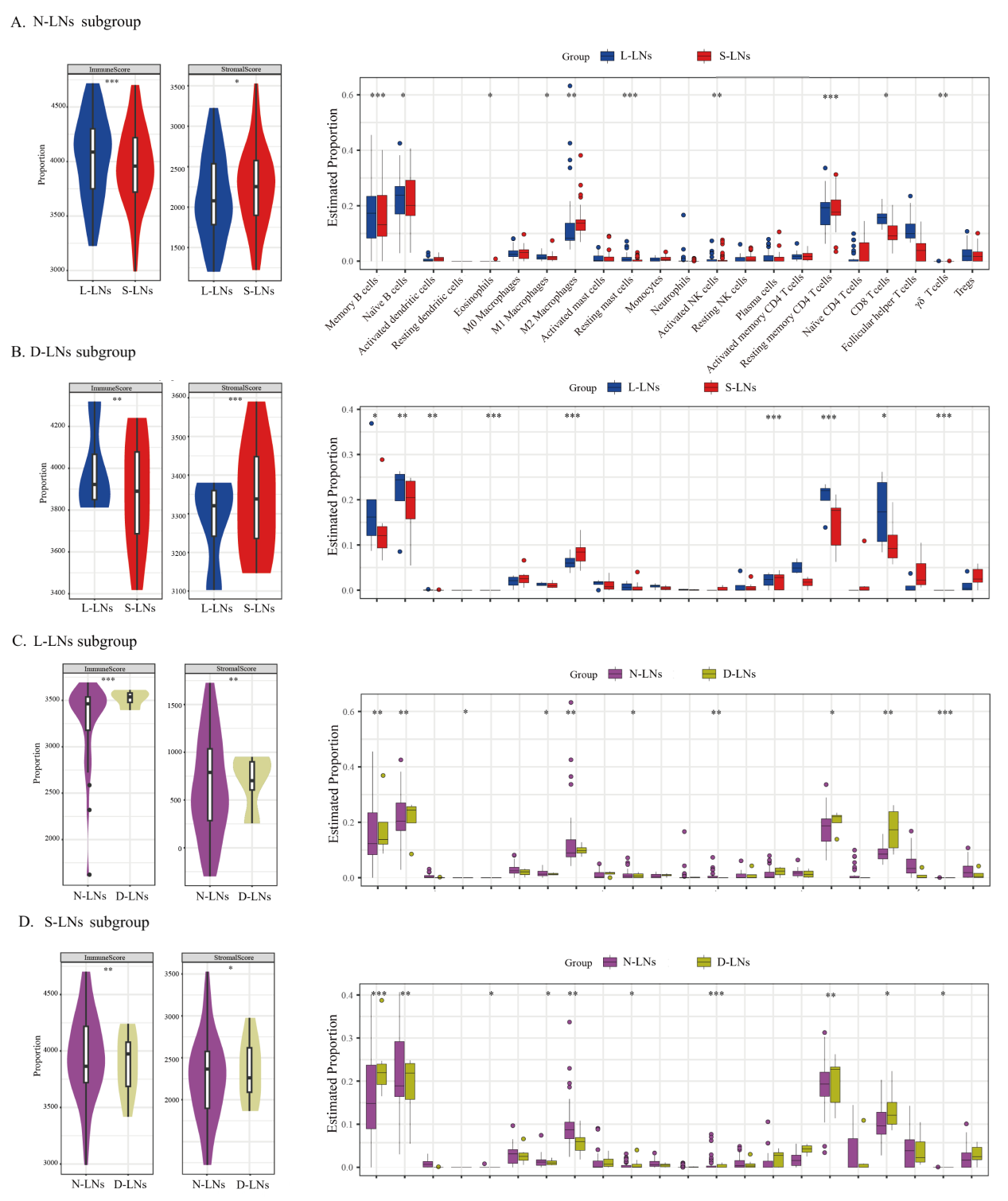
**

**Figure S7. Systems biology analysis of molecular pathways in LNs subgroups in prospective multimodal cohort. (A)** Gene set enrichment analysis (GSEA) identifies the pathways significantly enriched in L-LNs and S-LNs, highlighting distinct biological processes associated with each subgroup. **(B)** GSEA reveals the enriched pathways in D-LNs and N-LNs, providing insights into their functional differences at the molecular level. **(C-D)** Gene Ontology (GO) and Kyoto Encyclopedia of Genes and Genomes (KEGG) enrichment analysis of upregulated genes in L-LNs indicate significant enrichment in specific biological processes and pathways, while GO and KEGG analysis of upregulated genes in S-LNs reveal distinct pathway activation patterns. **(E-F)** GO and KEGG enrichment analysis of upregulated genes in N-LNs demonstrate enrichment in cancer-related pathways, suggesting their involvement in tumor progression. Conversely, GO and KEGG analysis of upregulated genes in D-LNs show a predominant enrichment in immune-related pathways, indicating a potential role in immune modulatio. S-LNs, small-LNs; L-LNs, large-LNs; N-LNs, near-LNs; D-LNs, distant-LNs.


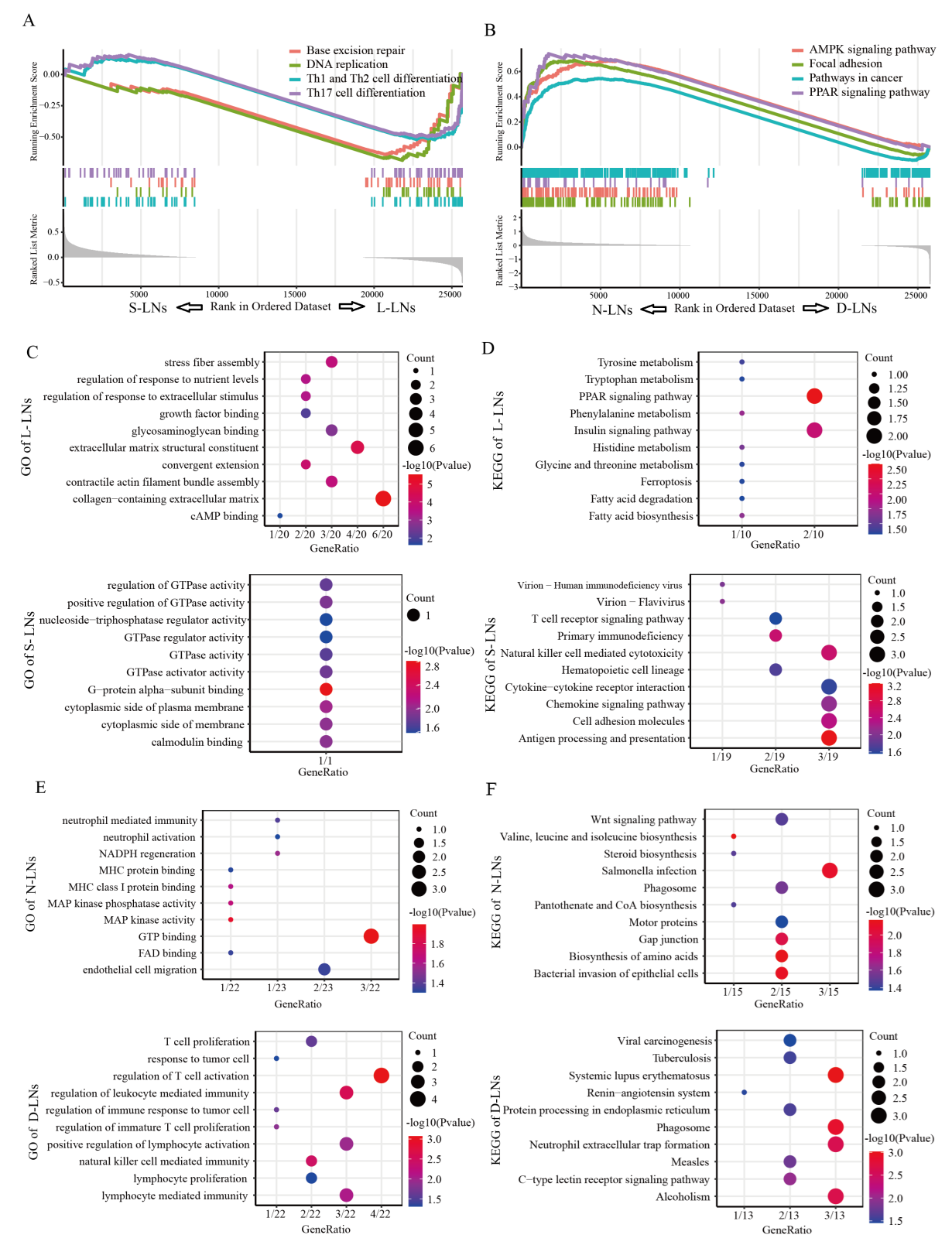


**Figure S8. Diagnostic performance evaluation of MRI-derived LNs imaging features across cohorts.** This figure presents ROC curve analysis assessing the predictive accuracy of tLNV and tLND. (A) Demonstrates the prognostic performance for overall survival prediction. (B) Evaluates disease-free survival prediction. The analysis spans all study cohorts (training, internal validation, and external validation), with each curve representing the sensitivity-specificity relationship at different cutoff thresholds. The consistent performance patterns across independent cohorts validate the robustness of these imaging features for clinical prognosis prediction. tLNV, total lymph nodes volume; tLND, total lymph nodes drainage distance.

**
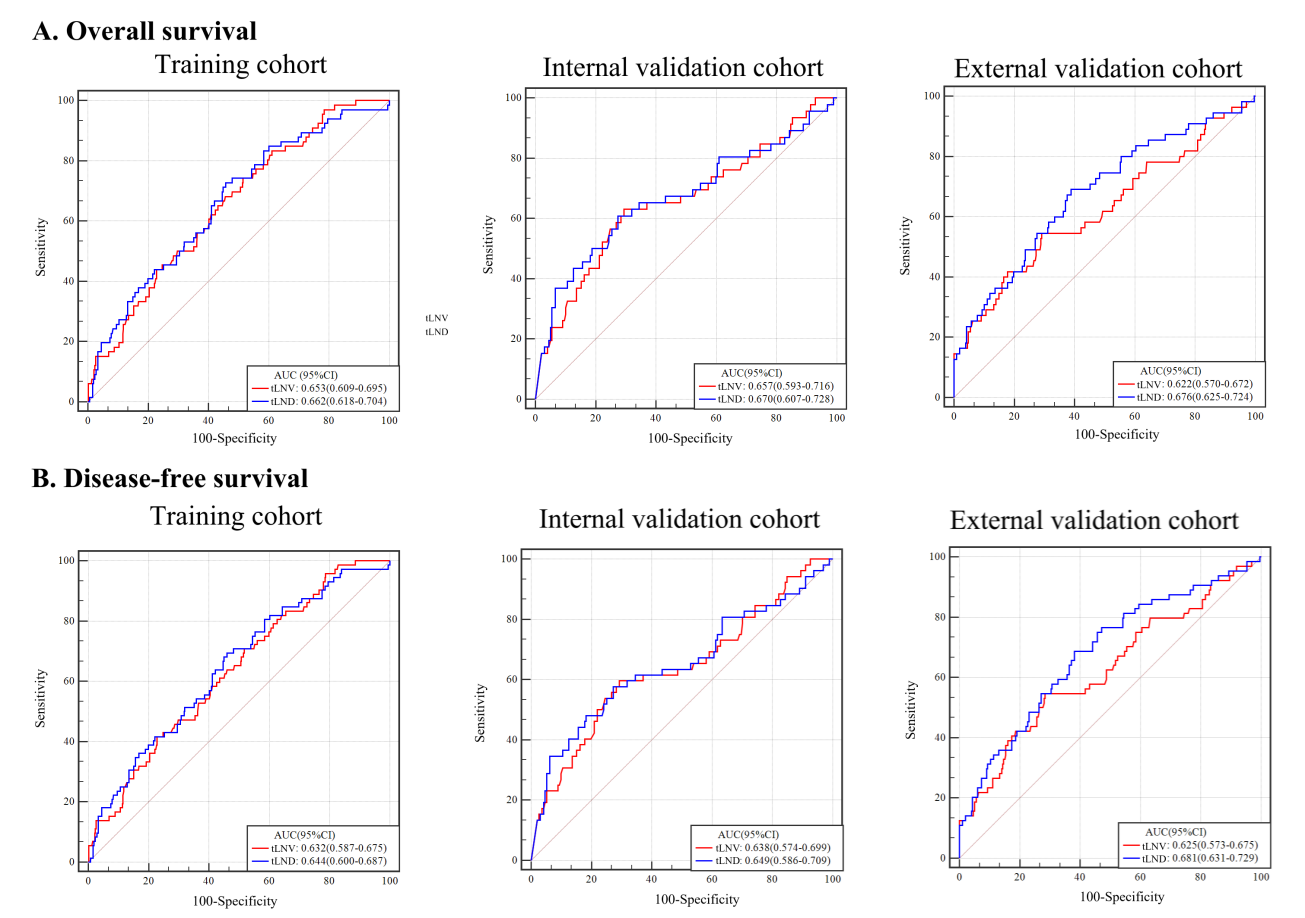
**

**Figure S9. Determination of optimal prognostic thresholds for MRI-derived LNs imaging features.** This figure illustrates the methodology for establishing clinically relevant cutoff values for the MRI-derived quantitative imaging features. The analysis identifies the optimal discrimination thresholds for tLNV and tLND through systematic evaluation of sensitivity and specificity trade-offs in the training cohort. tLNV, total lymph nodes volume; tLND, total lymph nodes drainage distance.

**
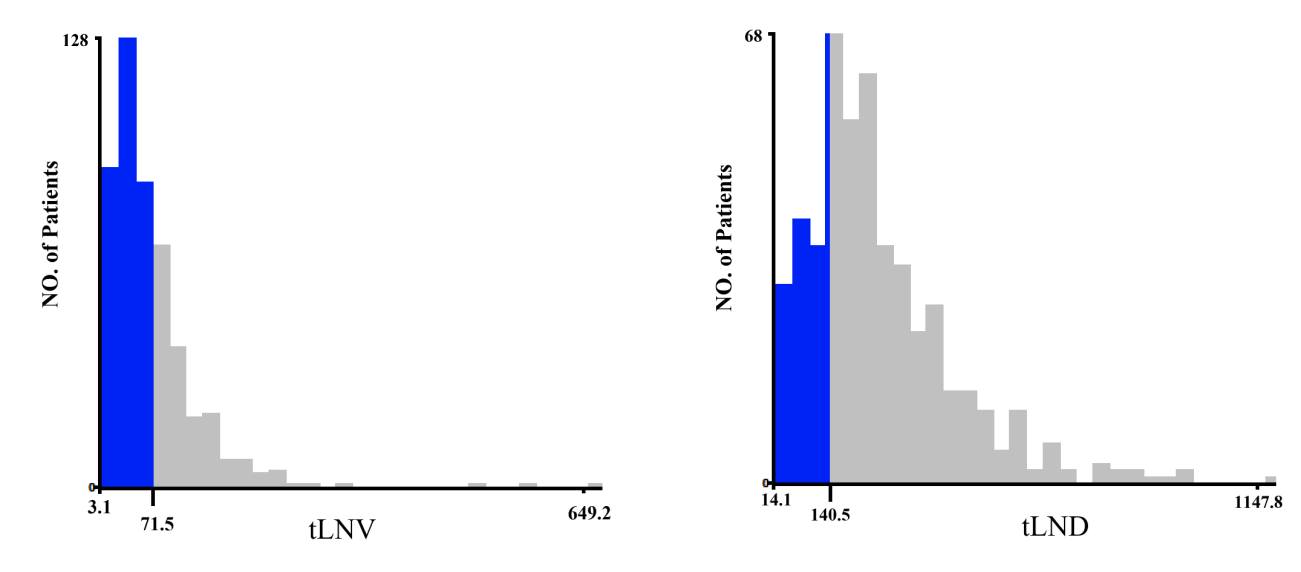
**

**Figure S10. Prognostic value of LNs imaging features for disease-free survival.** This figure demonstrates the consistent association between quantitative LNs imaging features (tLNV and tLND) and disease-free survival outcomes across all study cohorts. The Kaplan-Meier survival curves illustrate the significant stratification achieved by both tLNV and tLND in training cohort, internal and external validation cohorts. tLNV, total LNs volume; tLND, total LNs drainage distance; S-tLNV, small-tLNV; L-tLNV, large-tLNV; N-tLND, near-tLND; D-tLND, distant-tLND; HR, hazard ratio.

**
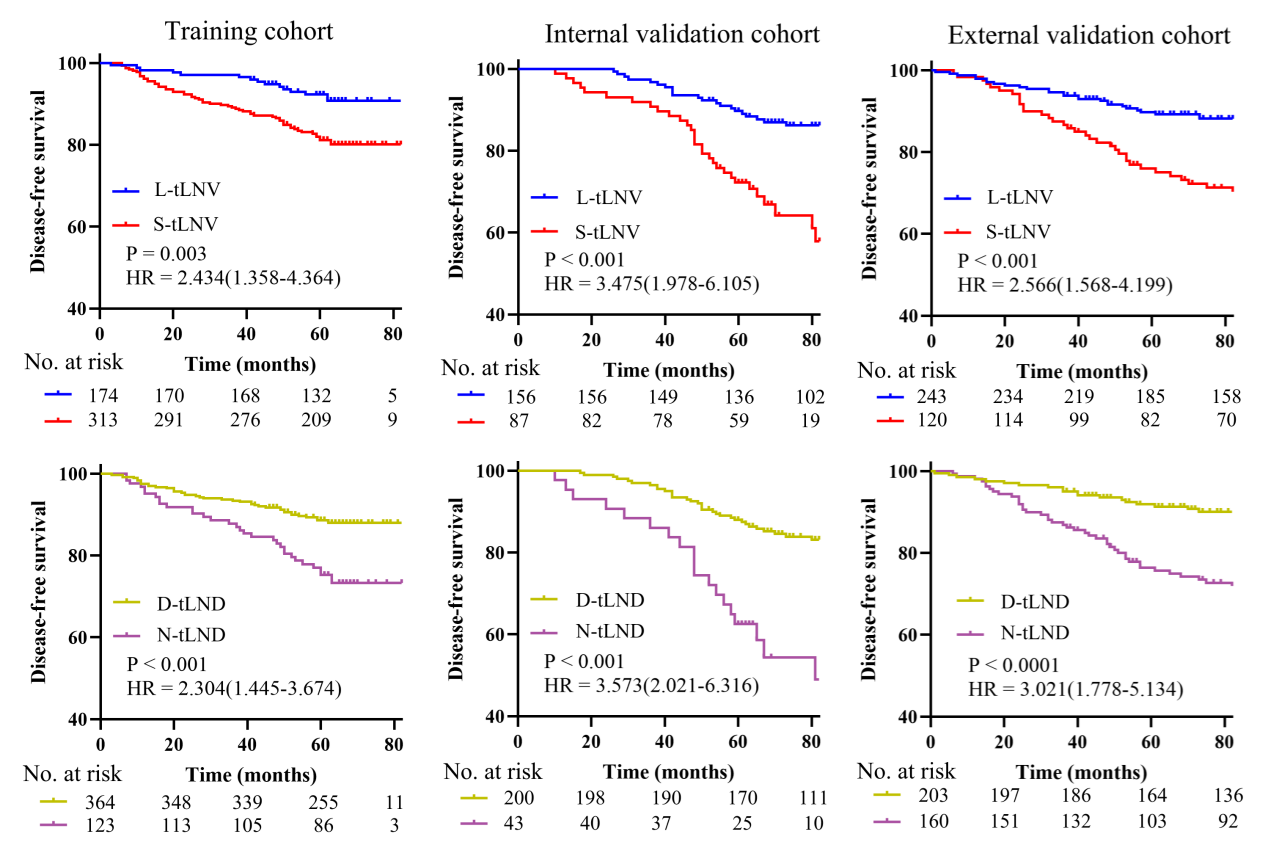
**

**Figure S11. Overall survival stratification by dichotomized tLNV across clinicopathological subgroups.** This figure presents Kaplan-Meier survival curves evaluating the prognostic significance of dichotomized tLNV across key clinicopathological subgroups. The analysis encompasses the entire study population (n=1093) from all three cohorts (training, internal validation, and external validation), demonstrating consistent discrimination between patients with S-tLNV versus L-tLNV. The stratified analysis reveal maintained prognostic value across varying patient characteristics, including age, tumor stages, and treatment modalities, etc. tLNV, total lymph nodes volume; S-tLNV, small-tLNV; L-tLNV, large-tLNV; HR, hazard ratio; LNE, lymph nodes examined.

**
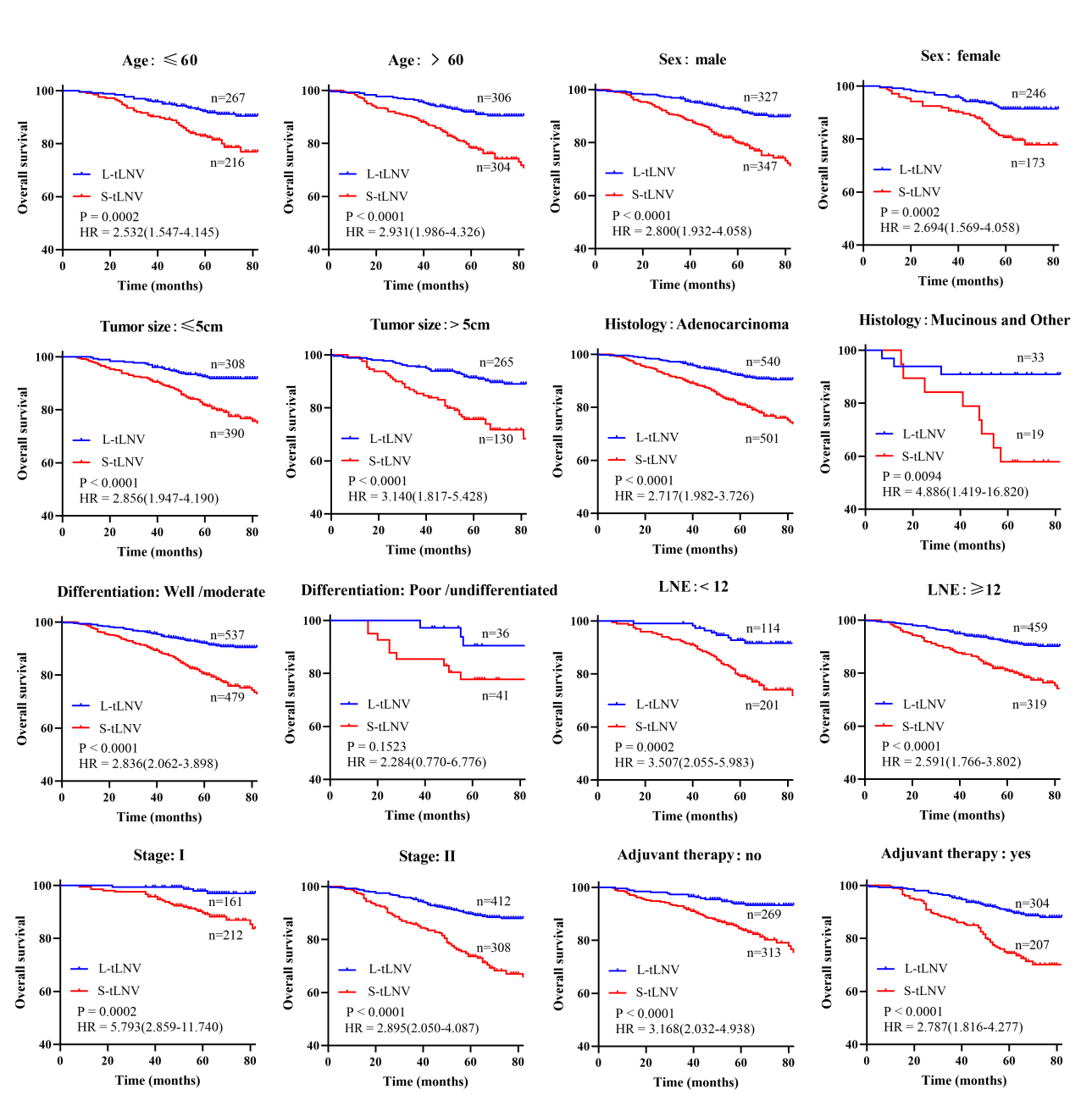
**

**Figure S12. Disease-free survival by dichotomized tLNV across clinicopathological subgroups.** This figure presents Kaplan-Meier survival curves evaluating the prognostic significance of dichotomized tLNV across key clinicopathological subgroups. The analysis encompasses the entire study population (n=1093) from all three cohorts (training, internal validation, and external validation), demonstrating consistent discrimination between patients with S-tLNV versus L-tLNV. The stratified analysis reveal maintained prognostic value across varying patient characteristics, including age, tumor stages, and treatment modalities, etc. tLNV, total lymph nodes volume; S-tLNV, small-tLNV; L-tLNV, large-tLNV; HR, hazard ratio; LNE, lymph nodes examined.


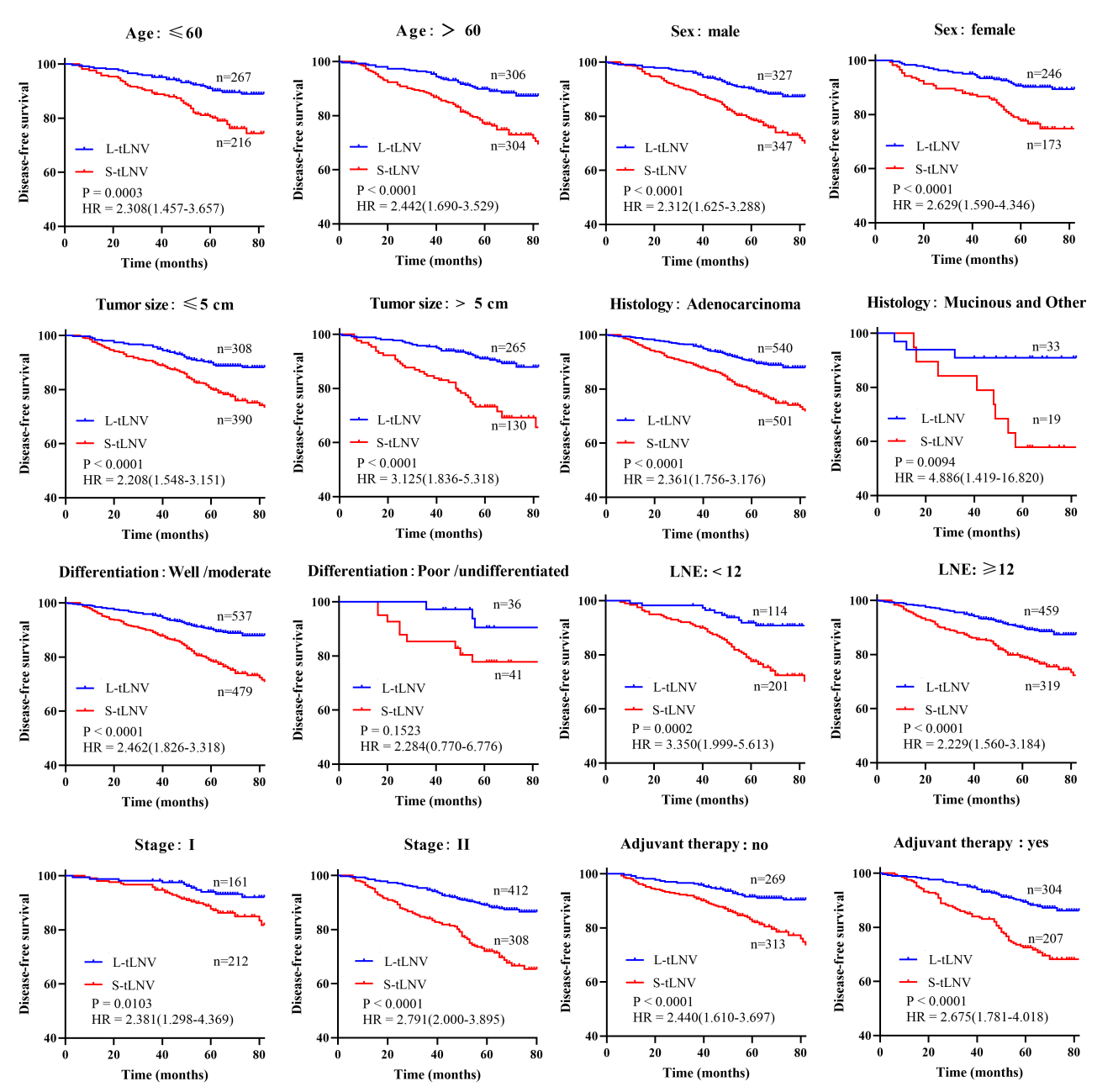


**Figure S13. Overall survival by dichotomized tLND across clinicopathological subgroups.** This figure presents Kaplan-Meier survival curves evaluating the prognostic significance of dichotomized tLND across key clinicopathological subgroups. The analysis encompasses the entire study population (n=1093) from all three cohorts (training, internal validation, and external validation), demonstrating consistent discrimination between patients with N-tLND versus D-tLND. The stratified analysis reveal maintained prognostic value across varying patient characteristics, including age, tumor stages, and treatment modalities, etc. tLND, total lymph nodes drainage distance; N-tLND, near-tLND; D-tLND, distant-tLND; HR, hazard ratio; LNE, lymph nodes examined.


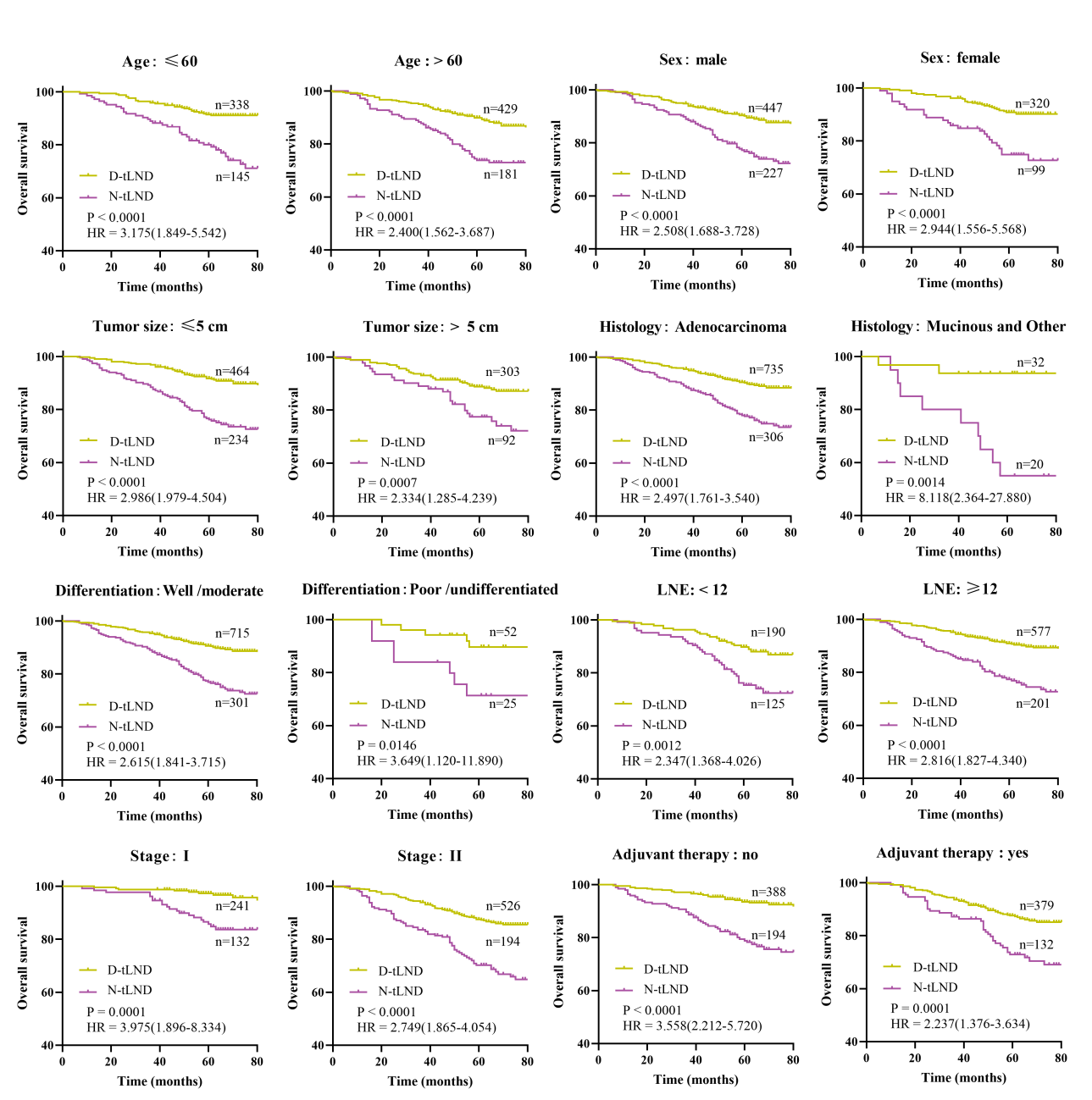


**Figure S14. Disease-free survival by dichotomized tLND across clinicopathological subgroups.** This figure presents Kaplan-Meier survival curves evaluating the prognostic significance of dichotomized tLND across key clinicopathological subgroups. The analysis encompasses the entire study population (n=1093) from all three cohorts (training, internal validation, and external validation), demonstrating consistent discrimination between patients with N-tLND versus D-tLND. The stratified analysis reveal maintained prognostic value across varying patient characteristics, including age, tumor stages, and treatment modalities, etc. tLND, total lymph nodes drainage distance; N-tLND, near-tLND; D-tLND, distant-tLND; HR, hazard ratio; LNE, lymph nodes examined.


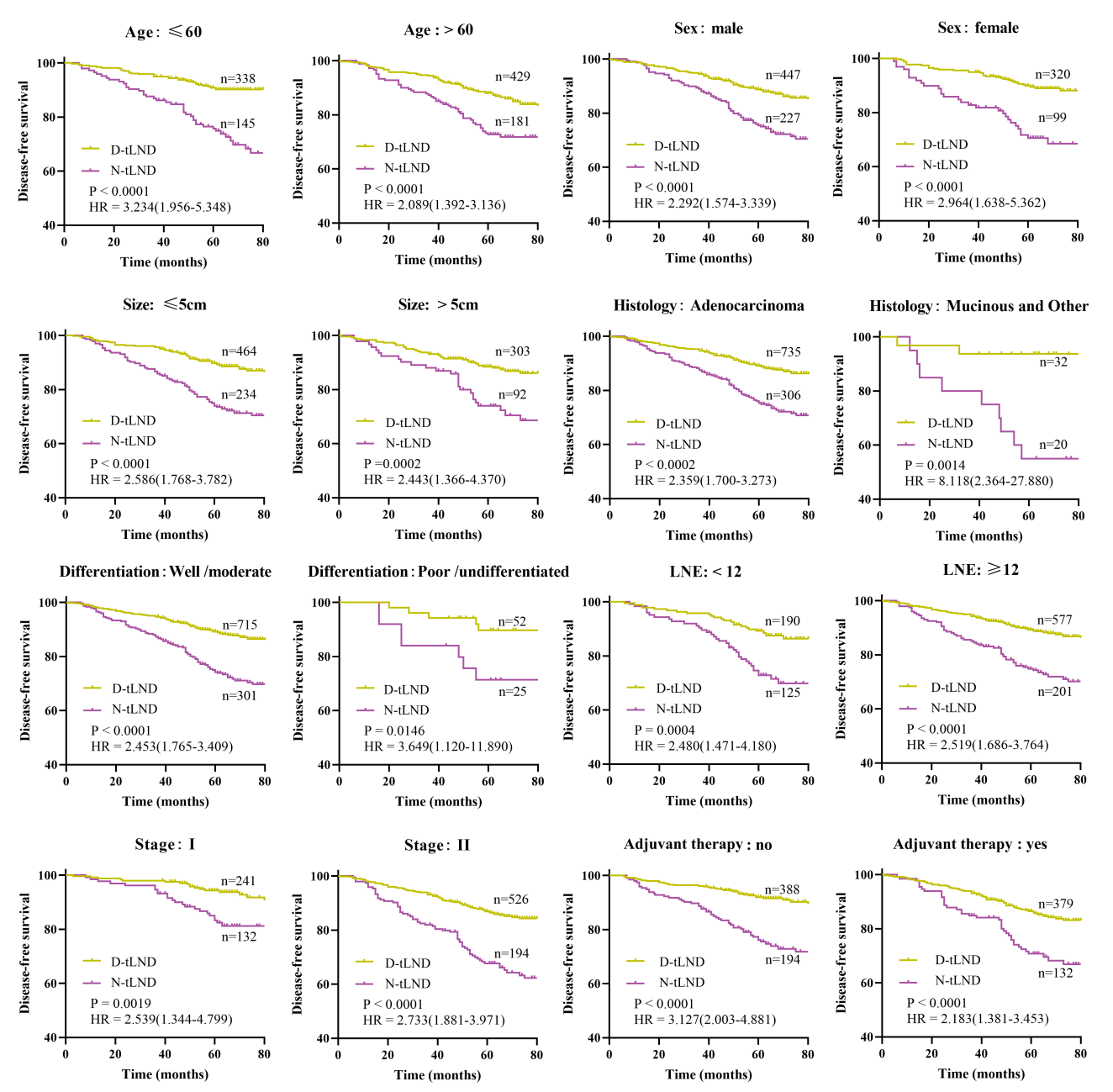


**Figure S15. Prognostic validation of LNs-MTS risk subtypes for overall survival.** This comprehensive analysis evaluates the clinical utility of the LNs-MTS risk stratification system (HRS, MRS, and LRS) in predicting overall survival outcomes for 1,093 patients from all three cohorts (training, internal validation, and external validation). The Kaplan-Meier survival curves demonstrate robust and consistent discrimination among all three risk subtypes, with the LRS showing the most favorable survival outcomes, followed by MRS and HRS. Importantly, this risk stratification maintains its prognostic significance when analyzed within key clinicopathological subgroups. HRS, high-risk subtype; MRS, moderate-risk subtype; LRS, low-risk subtype; HR, hazard ratio; LNE, lymph nodes examined.


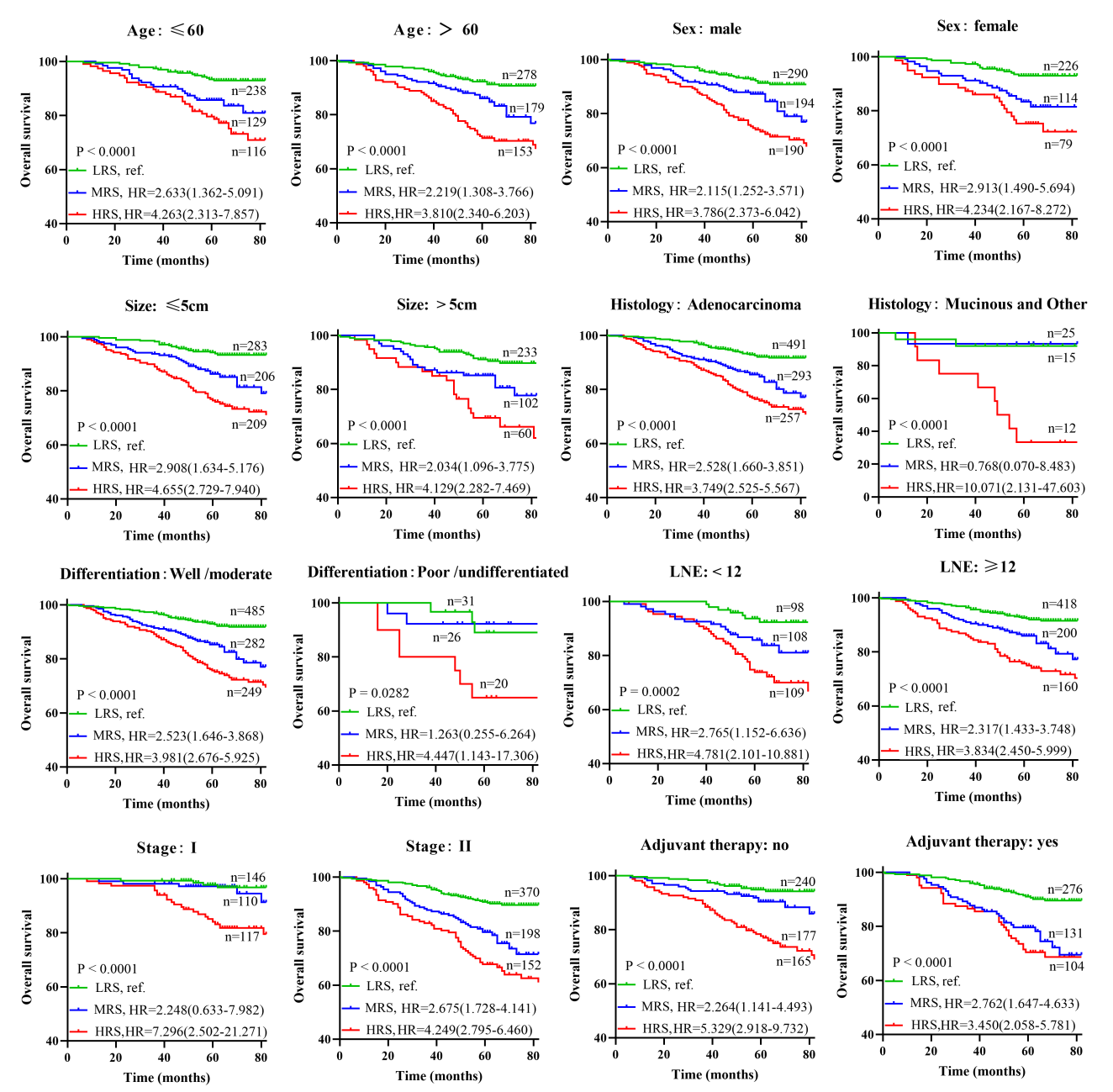


**Figure S16. Prognostic validation of LNs-MTS risk subtypes for disease-free survival.** This comprehensive analysis evaluates the clinical utility of the LNs-MTS risk stratification system (HRS, MRS, and LRS) in predicting disease-free survival outcomes for 1,093 patients from all three cohorts (training, internal validation, and external validation). The Kaplan-Meier survival curves demonstrate robust and consistent discrimination among all three risk subtypes, with the LRS showing the most favorable survival outcomes, followed by MRS and HRS. Importantly, this risk stratification maintains its prognostic significance when analyzed within key clinicopathological subgroups. HRS, high-risk subtype; MRS, moderate-risk subtype; LRS, low-risk subtype; HR, hazard ratio; LNE, lymph nodes examined.


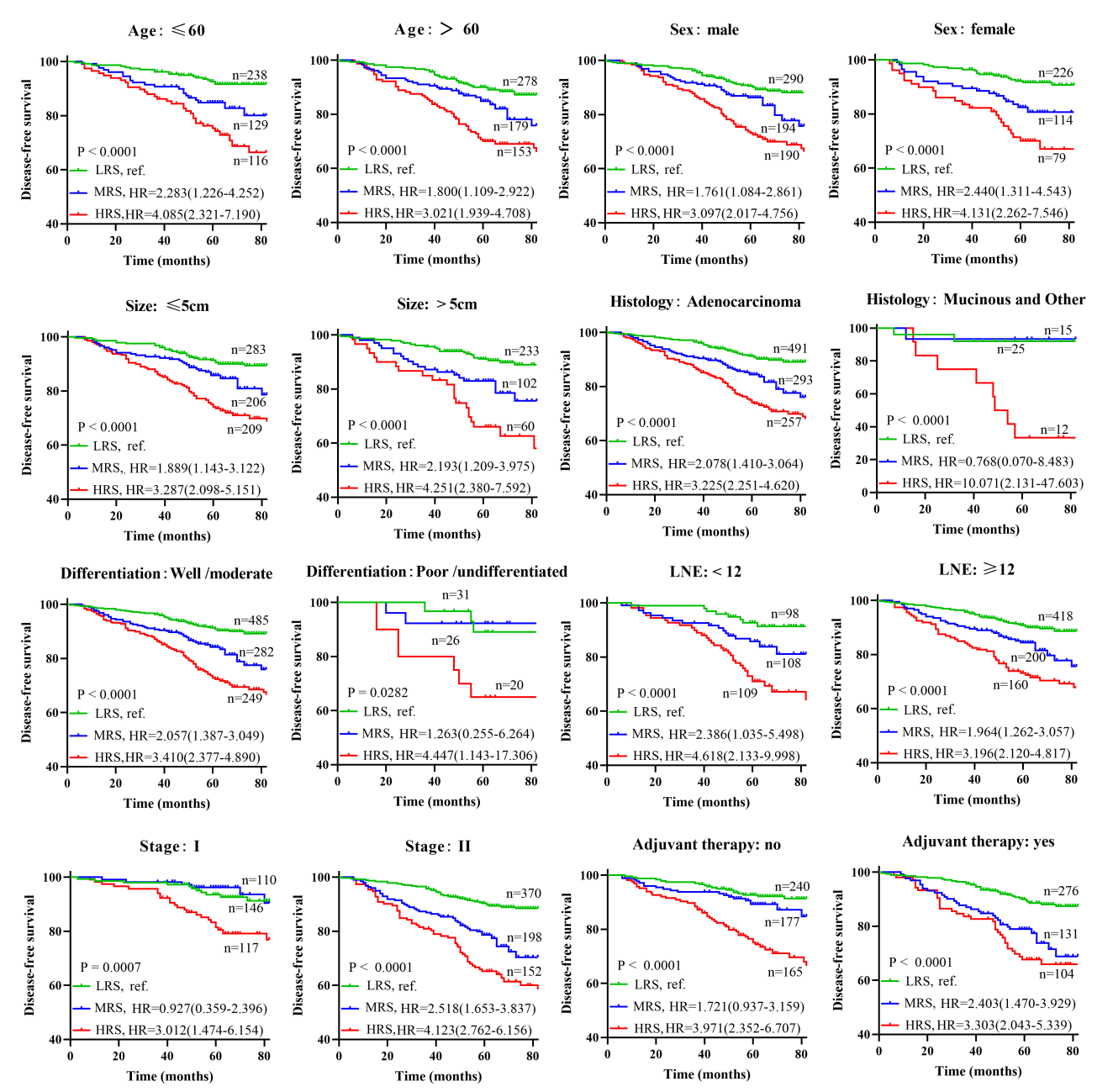


**Figure S17. Comparative prognostic performance of TNM staging system.** This analysis evaluates the relationship between guideline-based TNM staging system and clinical outcomes across all study cohorts. (A) Presents the overall survival analysis, demonstrating the prognostic discrimination achieved by current TNM staging criteria in both the training and validation cohorts. (B) Examines disease-free survival outcomes according to TNM staging, revealing similar stratification patterns. HR, hazard ratio.

**
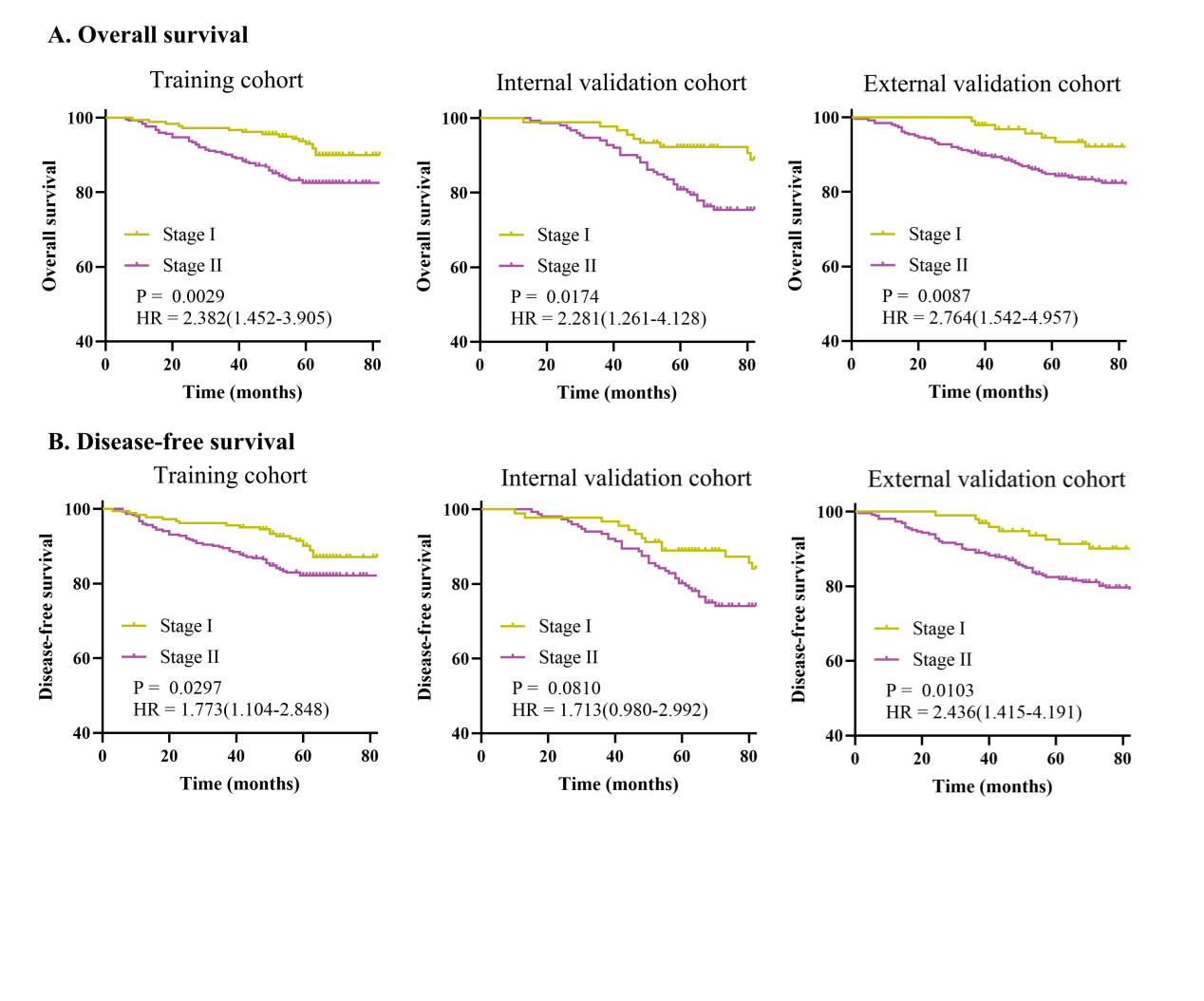
**

**Figure S18. Comparative prognostic performance of T staging system.** This analysis evaluates the relationship between guideline-based T staging system and clinical outcomes across all study cohorts. (A) Presents the overall survival analysis, demonstrating the prognostic discrimination achieved by current T staging criteria in both the training and validation cohorts. (B) Examines disease-free survival outcomes according to T staging, revealing similar stratification patterns; HR, hazard ratio.

**
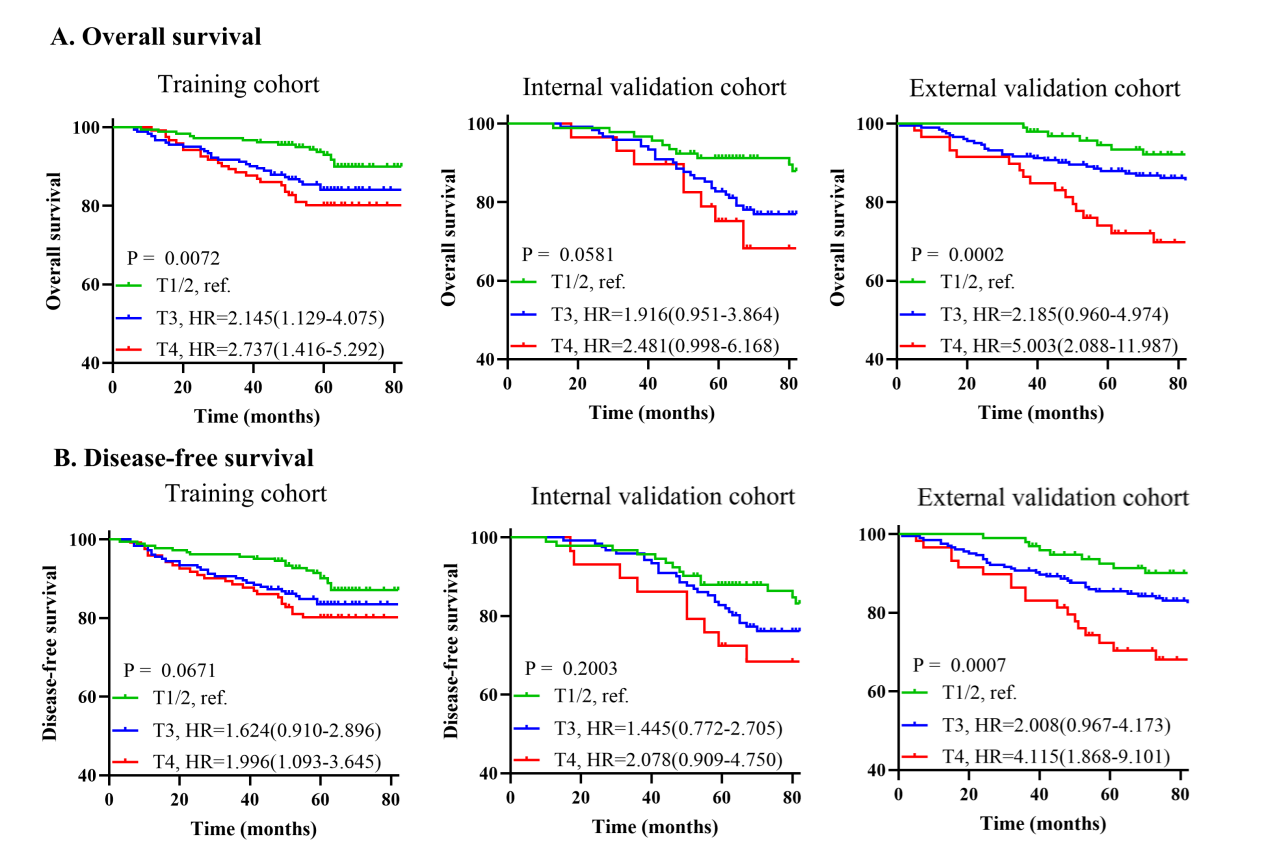
**

**Figure S19. Concordance analysis between LNs-MTS risk subtypes and guideline-based risk stratification.** The analysis reveals substantial heterogeneity within conventional stage categories, demonstrating how the LNs-MTS model identifies distinct prognostic subgroups that transcend traditional staging boundaries. Notably, the visualization highlights: (1) the presence of all three risk subtypes (LRS/MRS/HRS) within each TNM staging category, indicating additional prognostic refinement potential; (2) differential distribution patterns that may explain outcome variability within guideline-defined risk groups; and (3) clinically relevant reclassification of patients who might benefit from alternative management strategies. The proportion analysis provides quantitative evidence for the complementary value of LNs-MTS risk subtypes when used alongside existing staging systems. HRS, high-risk subtype; MRS, moderate-risk subtype; LRS, low-risk subtype.

**
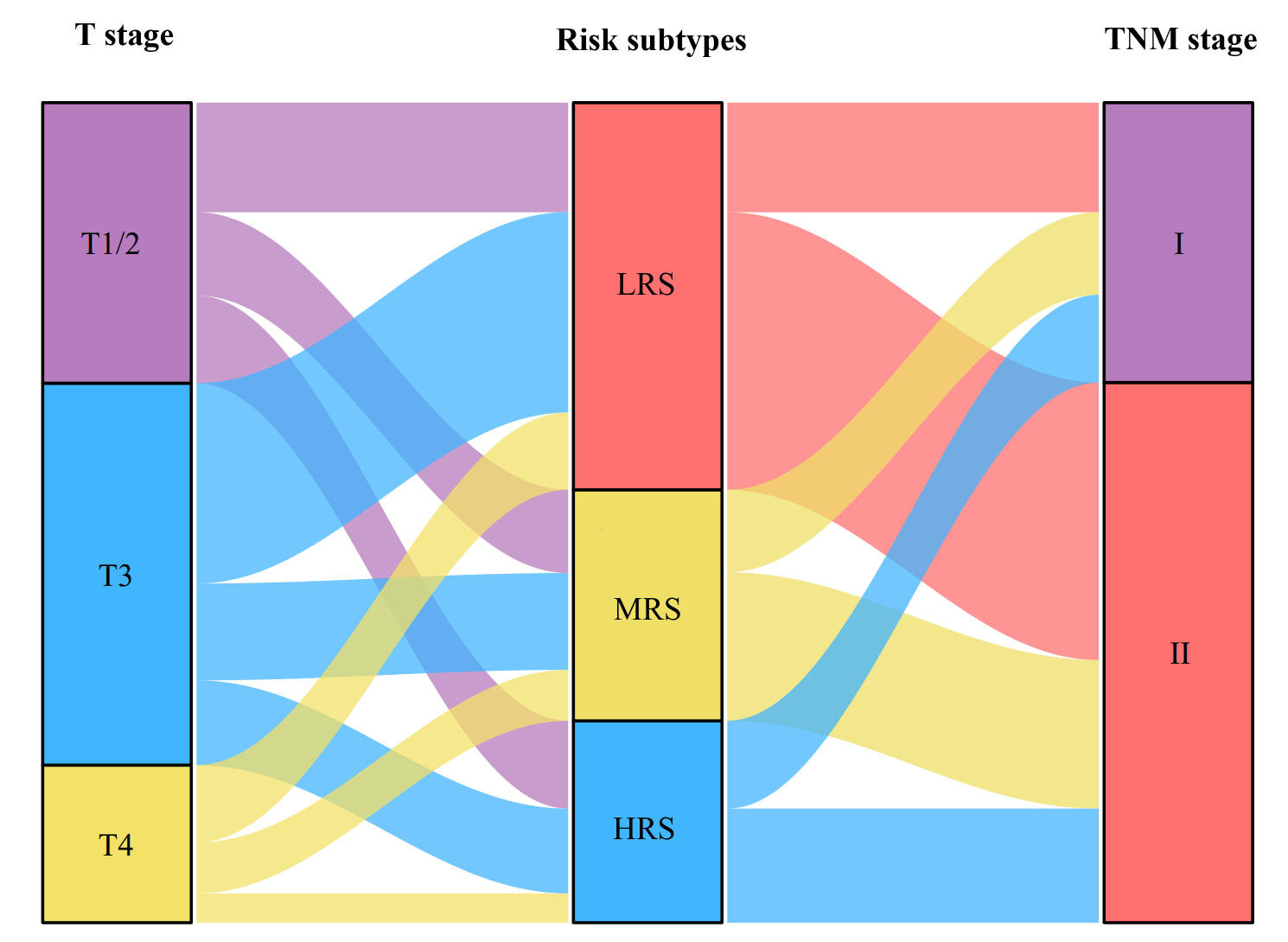
**

**Figure S20. LNs-MTS guided adjuvant therapy decision framework for early-stage rectal cancer.** This figure presents a clinically actionable algorithm for postoperative treatment stratification based on the integration of TNM staging with LNs-MTS risk subtypes. The proposed decision matrix addresses two key clinical challenges in early-stage rectal cancer management: (1) identification of high-risk stage I patients who may benefit from adjuvant therapy despite favorable conventional staging, and (2) recognition of low-risk stage II patients who could potentially avoid unnecessary treatment. HRS, high-risk subtype; MRS, moderate-risk subtype; LRS, low-risk subtype; RC, rectal cancer.


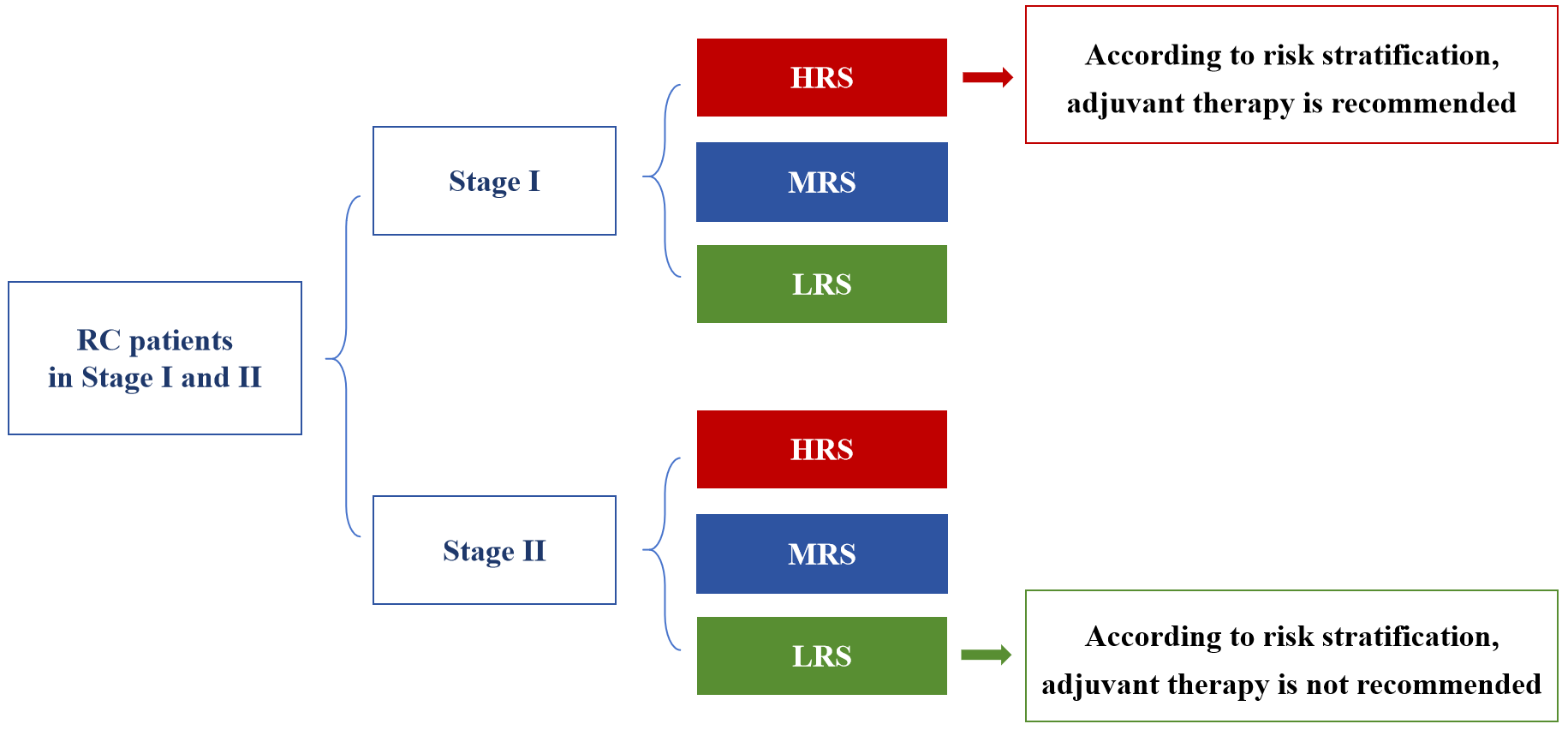


**Figure S21. Determination of optimal classification thresholds for LNs size and spatial distribution.** (A) For size stratification, systematic evaluation of immune marker expression profiles across a clinically relevant size spectrum (0.2-1.0 cm) identified 0.5 cm diameter as the optimal threshold, effectively differentiating immunologically active L-LNs (≥0.5 cm) from S-LNs (<0.5 cm). (B) For distance stratification, analysis of immune marker differential expression across varying distances (2-10 cm) from the primary tumor revealed 5 cm as the most discriminative boundary, separating D-LNs (≥5 cm) with preserved immune function from N-LNs (<5 cm) showing tumor-mediated immunosuppression. S-LNs, small-LNs; L-LNs, large-LNs; N-LNs, near-LNs; D-LNs, distant-LNs.

**
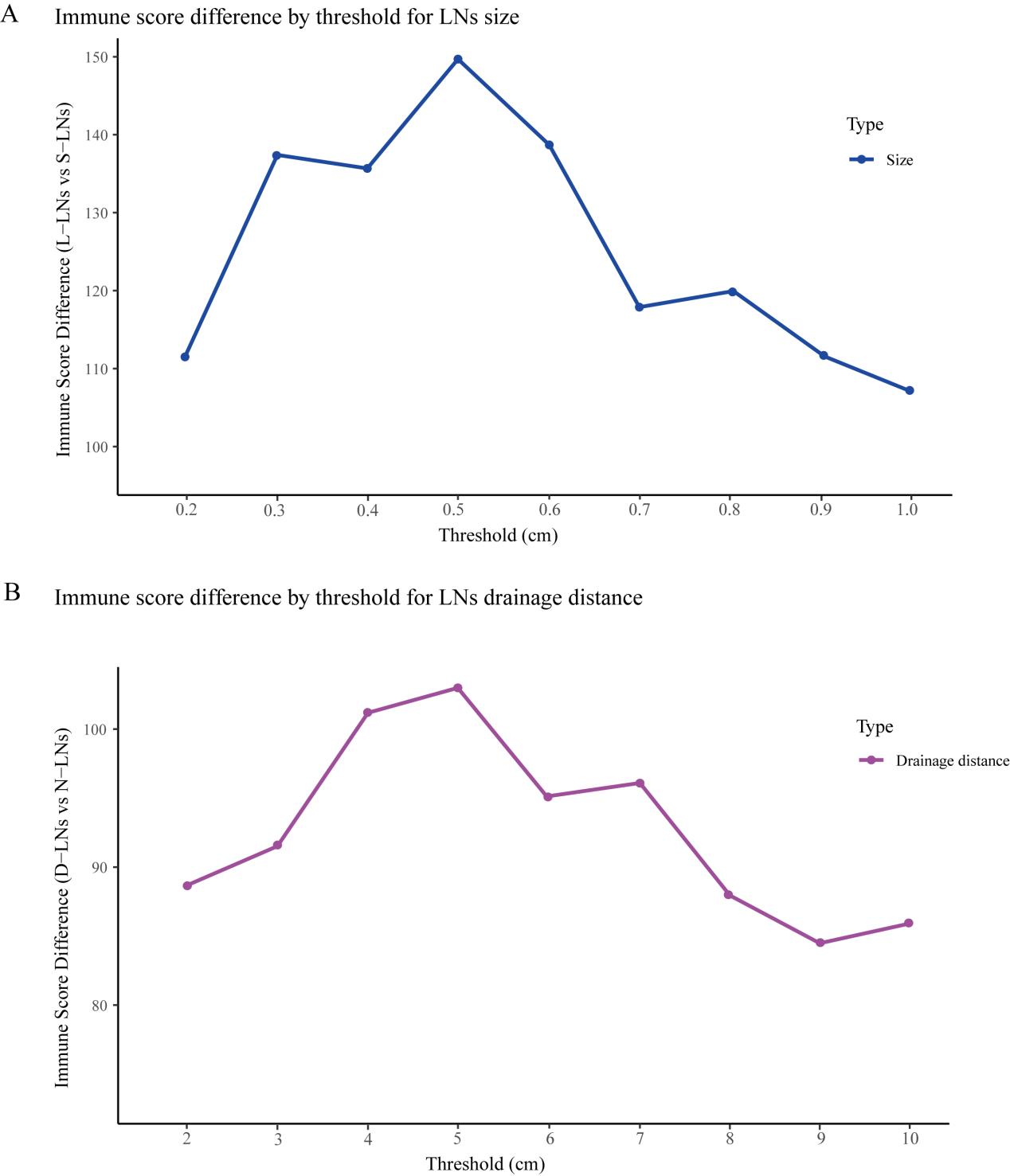
**
